# Supplementary material for: Barriers and facilitators to antibiotic stewardship in Nigeria’s private healthcare sector: A qualitative interview study with national health and regulatory interest holders
Source: PLOS Glob Public Health. 2026 Jan 6;6(1):e0005731. doi: 10.1371/journal.pgph.0005731 (PMC12774337; doi:10.1371/journal.pgph.0005731)
Supplement: S2 File — (PDF) [file pgph.0005731.s002.pdf]

S2 File – Interview guide

1. Please tell me about your **role**.
   Probe: position /role; what you do; what are your defined responsibilities; how long have you worked at your current organization.

2. Please tell me what you **know**, or have heard, about antimicrobial resistance, especially in developing nations, or in Africa?
   Probe: 1) What can you say about antimicrobial resistance in Nigeria? 2) The West Africa sub-region is said to have the highest prevalence in Africa and Nigeria constitutes almost 50% of the West African population. Any comment? 3) Have you heard of the WHO AWaRe handbook?

3. Does your organization play any **role** (directly or indirectly) in *antimicrobial stewardship* (defined as: Designing and implementing initiatives to reduce and improve antibiotic prescribing practices)?
   Probe: What kind of role?

4. What do you perceive as the role of the private sector in antimicrobial stewardship (AMS) in Nigeria?
   Probe: In your opinion, are private sector providers contributing to the problem or to solutions for ensuring rational drug use? In what way? Are you aware of any AMS activities in the private sector?

5. What do you think are some of the **barriers** to implementing an antimicrobial stewardship intervention that targets the private sector, and particularly primary care physicians operating in this sector?
   Probe: 1) Problems interacting with private sector, 2) problems regulating provider practices, 3) community-related barriers to antimicrobial stewardship.

6. What are some of the **opportunities** for implementing interventions or actions targeting the rational use of antimicrobial agents?
   Probe: 1) Priority areas (from clinicians' prescribing practices to dispensing by pharmacists/medicine vendors), 2) Opportunities for improving provider knowledge, attitudes, skills, and behavior, 3) Feasibility aspects (e.g., access to antimicrobial susceptibility testing, provider training), 4) Acceptability aspects (how well received do you think these are or would be among primary healthcare providers?).

7. What are some of the **challenges** to implementing interventions or actions targeting the rational use of antimicrobial agents?
   Probe: 1) Priority areas (from clinicians' prescribing practices to dispensing by pharmacists/medicine vendors), 2) Challenges associated with provider knowledge, attitudes, skills, and behavior, 3) Feasibility aspects (e.g., access to antimicrobial susceptibility testing, provider training), 4) Acceptability aspects (how well received do you think these are or would be among primary healthcare providers?).

8. What do you think could be done to improve current practices related to antimicrobial use, generally and specifically among primary healthcare providers in the private sector?
   Probe: Where are the identified leakages that need to be blocked to improve antimicrobial stewardship in the country? What are some key areas for improvement and your recommendations?

9. Please tell me any other thing you would like to share about difficulties or facilitators to implementing an antimicrobial stewardship intervention among private primary healthcare practitioners.
   Probe: 1) Barriers and challenges to antimicrobial stewardship interventions? 2) Facilitators of antimicrobial stewardship interventions?
