# Supplementary material for: Barriers and facilitators to antibiotic stewardship in Nigeria’s private healthcare sector: A qualitative interview study with national health and regulatory interest holders
Source: PLOS Glob Public Health. 2026 Jan 6;6(1):e0005731. doi: 10.1371/journal.pgph.0005731 (PMC12774337; doi:10.1371/journal.pgph.0005731)
Supplement: S3 File — (PDF) [file pgph.0005731.s003.pdf]

S3 File – Themes and Subthemes

Theme #1: Role of Private Sector

- The private sector as a key player in Nigeria's health sector
    - Interview 1
        - "Greater role of the private sector, referral to public, tertiary if no solution"
        - *"Private sector plays a crucial role in healthcare; majority of Nigerians (70–80%) pay out of pocket for medical services."*
        - *"Private sector plays a key role; improving prescription practices and rational antimicrobial use can set a strong example."*
    - Interview 2
        - *Public sector healthcare is not optimal; many rely on private sector care paid out of pocket.*
    - Interview 3
        - "Private sector has a key role in addressing non-standard prescription practices."
        - "Private sector includes both clinicians and non-clinicians; some health workers run small practices and prescribe based on patient affordability rather than proper protocols."
        - "Some private sector facilities follow tertiary institution standards; consultants maintain proper prescriptions; pharmacists ensure correct medications and dosing."
        - "Well-regulated private facilities pose fewer issues; profit-driven facilities without pharmacists allow health workers to administer suboptimal antimicrobial doses."
    - Interview 5
        - Private sector plays a crucial role in AMR response; most Nigerians prefer private healthcare due to gaps in the public system; majority of antimicrobial use occurs in private facilities
        - Private sector is vital in all strategic objectives of global and national AMR action plans, not just stewardship
        - At least 70% of Nigerians prefer private healthcare, where most antimicrobials are consumed; need

stronger private sector involvement in stewardship and data use

- Interview 6
    - Primary healthcare is the foundation of healthcare, responsible for 70% of the population; plays a crucial role as the first point of contact for healthcare in Nigeria.
- Interview 8
    - Role of the Private Sector in Healthcare; Private sector is more accessible to communities than public tertiary institutions; Operates at the primary and secondary levels.
    - Role of Tertiary Institutions and Private Sector; Tertiary institutions are primarily teaching hospitals; Private sector provides healthcare for about 60% of the population
    - ealthcare Utilization Trends; Over 60% of Nigerians seek care in the private sector for illnesses and medications
    - Healthcare Utilization Trends; 60% of the population seeks healthcare in the private sector; Private sector plays a key role in health service delivery
    - Private Sector Dominance in Healthcare; Primary and secondary healthcare facilities are mostly privately operated; Private institutions play a major role in healthcare
    - Healthcare Referral System in Nigeria; Cases move from primary to secondary to tertiary healthcare; Structured referral system guides patient care escalation.
    - Private Sector Commitment to Quality Healthcare; Private facilities serve over 60% of the population; Strive to provide high-quality healthcare services
    - Targeting AMR Stewardship Efforts; Private sector engagement improves patient wellness; Presents a promising area for antimicrobial stewardship initiatives
- Interview 9
    - Role of Private Sector in AMR Efforts; Private sector plays a key role in combating antibiotic resistance; Cares for over 60% of patients requiring antibiotics in Nigeria
- Interview 13

        - "Private Healthcare Facilities in Nigeria; Private Sector Providing 60% of Healthcare Services; Public Sector Providing 40% of Healthcare Services."

- Barriers to private sector engagement in AMR response
    - Interview 1
        - "Private sector perceives government audits as fault-finding; skepticism about prescription audits; some resistance exists, but mitigated through strategic approach."
    - Interview 2
        - Funding barriers for private facilities; nonprofit funders avoid supporting profit-driven organizations; concern over using taxpayer money for private businesses.
        - MOUs needed to ensure private sector funding is not used for profit; legal agreements could help private facilities access AMR program funding; lack of such structures is a challenge.
    - Interview 3
        - "Some private tertiary centers follow standard treatment protocols; many private facilities lack clinician oversight."
    - Interview 5
        - Limited data on antibiotic use in the private sector, but a significant portion of antimicrobials are used there
        - Limited antimicrobial stewardship programs in the private sector; most efforts focus on 30-40 public healthcare facilities within specific states
        - AMR partners work directly with states and communities, not at the national level; national technical working group for stewardship exists, but private sector involvement remains limited
        - Limited antimicrobial stewardship programs in the private sector; most efforts focus on 30-40 public healthcare facilities within specific states
        - "AWARE classification needs strengthening at sub-national, tertiary, and secondary levels; healthcare facilities should implement antibiotic stewardship."
    - Interview 6
        - Organization's mandate is focused on primary healthcare facilities, not the private sector; state-level involvement may be limited in engaging the private sector directly.

    - Private sector challenges include lack of laboratory facilities for diagnostic tests and antimicrobial sensitivity tests, hindering effective treatment and contributing to resistance.
    - Private establishments at the engagement level are not conducting antimicrobial sensitivity tests, which limits proper AMR management and effective treatment
- Interview 8
    - Limited AMR Stewardship Awareness; Few healthcare facilities implement AMR stewardship; Awareness and adoption rates remain low.
    - "Healthcare Access in Nigeria; Over 60% of patients access care in the private sector; Government hospitals alone cannot meet the healthcare needs of Nigeria's 200 million+ population."
- Interview 10
    - "Unregulated Access to Antimicrobials; Widespread availability of antibiotics without prescriptions; Lack of proper microbiology investigations before use."
    - "AMR as a Hospital and Societal Issue; Widespread antibiotic abuse leading to resistance; Significant problem in both hospitals and the broader society in Nigeria."
    - "Challenges in Communication; Poor communication affects antimicrobial stewardship; Need for better messaging to stakeholders."
    - Failure to Follow National Guidelines; Doctors often skip investigations before prescribing antimicrobials; National guidelines from NAFDAC and NCDC exist but are not strictly followed
- Interview 11
    - "Challenges in SOP Implementation in Private Sector; Limited implementation of SOPs in private institutions; Few private healthcare providers in the region follow proper SOPs."
    - "Need for Proper SOPs and Infrastructure in Private Sector; Private institutions should have SOPs, appropriate cultural mediums, and proper environments for microbial testing."
    - "Proper SOPs should include correct mediums, environment, and conditions for microbial testing;

    Addressing infrastructure gaps in private institutions can help mitigate AMR risks."
  - "Challenges in SOP Implementation in Private Sector; Limited implementation of SOPs in private institutions; Few private healthcare providers in the region follow proper SOPs."
  - "Need for Proper SOPs and Infrastructure in Private Sector; Private institutions should have SOPs, appropriate cultural mediums, and proper environments for microbial testing."
- Interview 12
  - "Primary Healthcare System in Nigeria; Overburdening of Tertiary Healthcare; Role of Primary Care in Reducing Self-Medication; Need for Proper Referral Systems."
  - "Weak Primary and Secondary Healthcare Systems; Overburdened Tertiary Healthcare; Healthcare System Dysfunction in Nigeria."
- Interview 13
  - Greater Regulation in Public Health Institutions; Limited Regulation in Private Sector; Impact of Large Numbers in Public Healthcare.
  - Private Sector Engagement in Antimicrobial Resistance (AMR); Limited Attendance at AMR Meetings; Participation Through Grand Rounds and Seminars; Involvement in Policy Development.
  - "Lack of Equipment and Training in Primary Healthcare Centers; Limited Knowledge and Skills in Antimicrobial Resistance (AMR); Barrier to Effective Healthcare Delivery."
- Interview 14
  - "AMR Stewardship in Private vs. Public Sector; Lack of Organization in Private Sector Stewardship; Support from International Organizations for Government Hospitals."
  - "Progress in AMR Stewardship in Government Hospitals; Limited Progress in Private Sector; Ongoing Needs for AMR Improvements in Private Healthcare."
  - "Greater Focus on AMR Stewardship in Public Hospitals; Limited Progress in Private Sector Stewardship Efforts."
  - "Challenges in Restricting Antibiotic Use; Difficulty Implementing Restriction Lists Across Hospitals; Need

for Better Understanding Among Healthcare Professionals."
            - Challenges in Private Sector Antibiotic Treatment; Overuse of Presumptive Antibiotics; Lack of Local Data on Resistance Patterns; Limited Lab Capacity
            - Lack of Approval Requirements for Senior Clinicians Prescribing Restricted Antibiotics; Increased Frequency of Point Prevalence Audits; Development of Treatment Guidelines in Hospitals

- Facilitators of AMR stewardship in the private sector
    - Private Sector Assets
        - Interview 2
            - "No resistance from private providers; science is based on reasoning; fair acceptability of counseling and awareness initiatives"
        - Interview 4
            - Building capacity in the private sector is the top priority; ensuring effective laboratory diagnosis is key
            - Awareness is not low; increasing awareness in major institutions can further strengthen AMR prevention efforts
        - Interview 5
            - Technology transfer and pre-qualification processes for local pharmaceutical production have started; unclear how the private sector is involved
        - Interview 7
            - Private sector; Higher patient literacy, less abuse of antimicrobials, better adherence to treatment
            - Private sector; Better adherence and practices due to higher literacy levels among patients
            - "Private sector; Easier communication with patients, conducive environment for interaction, challenges with some patients.
            - Private vs. Public sector; Public sector, high patient volume, limited interaction; Private sector, better care, communication, and attention
        - Interview 8
            - Private Sector Majority in Healthcare; Majority of primary and secondary healthcare facilities are privately owned; Presents an opportunity for targeted AMR intervention.

        - Willingness of Private Sector to Adapt; Private healthcare providers are open to new information; Receptive to initiatives that improve healthcare provision
        - Targeting AMR Stewardship Efforts; Private sector engagement improves patient wellness; Presents a promising area for antimicrobial stewardship initiatives
      - Interview 10
        - "Issue is not tied to specific cultural groups; General agreement that seeking medical care is necessary when unwell."
      - Interview 11
        - Private Healthcare Sector's Role in AMR Prevention; Engaged in partnerships with the State Government and private institutions; Actively involved in healthcare and policy efforts
      - Interview 13
        - Involvement of Private Sector in Antimicrobial Resistance (AMR); Deficiency in Monitoring and Regulation of Private Healthcare; Public Institutions Playing a Larger Role in Regulation.
        - Capacity Building in Private Sector for Antimicrobial Stewardship; Grand Rounds, Seminars, Conferences, and Peer Review Meetings in Health Facilities
      - Interview 14
        - Opportunity from COVID-19 Pandemic; Recognition of Private Sector as Key Healthcare Stakeholder
        - Government-Private Sector Collaboration During COVID-19; Successful Model for Wide-Scale Testing.
        - Government-Private Sector Collaboration in Health; Expanding Reach Through Private Facilities and Laboratories; Ongoing Discussions on Engaging Private Sector in AMR Policy
        - Applying COVID-19 Certification Model to AMR; Government Support and Training for Private Labs; Enrollment in National Antimicrobial Stewardship Network.
  - Institutional Engagements
    - Nigerian organizations, including NCDC, WHO, NAFDAC, and medical councils, coordinate AMR response, regulate antimicrobial use, and promote stewardship initiatives.
      - Interview 1

    - "NAFDAC's role in reporting rare reactions, investigating genuine drugs, some antibiotics are not controlled or regulated"
- Interview 2
    - Nigeria CDC serves as National Coordinating Center for AMR response and long-term activities to prevent unnecessary use of antimicrobials.
- Interview 4
    - The Department of Hospital Services oversees the Medical Science Laboratory division; coordinates lab activities under medical lab scientists; WHO-supported initiatives included
    - Each institution has an Infection Prevention and Control Committee that develops prescription guidelines; stepwise approach prioritizes simpler antibiotics before stronger ones
- Interview 5
    - WHO supports procurement and supply chain of antibiotics, vaccines, and diagnostics; committee member assisting in guideline implementation and development
    - WHO is a key technical agency in Nigeria's AMR response; provided technical and financial support for developing the National Action Plan over the last two years
- Interview 6
    - "Nigeria adapts, rather than adopts, WHO AMR guidelines; follows global best practices and gold standards based on knowledge, research, and international experience."
    - National Primary Development Agency in addressing AMR challenges
    - NCDC is responsible for antimicrobial stewardship in Nigeria, coordinating efforts and collaborating with partners to promote optimal use of antimicrobial agents.
- Interview 8
    - Organizational Involvement; Leading role in Nigeria's human health component of the Fleming Fund; Organizational engagement in antimicrobial resistance efforts

    - Organizational Focus and Drug Resistance; Institute focuses on HIV, TB, malaria, and cancer; Resistance to common anti-TB drugs is a growing issue
- Interview 9
    - AMR Management within the Federal Ministry of Health; A dedicated department handles antimicrobial resistance; Interviewee’s office is not directly responsible but coordinates efforts
    - Federal Ministry of Health Oversight; Oversees both public and private hospitals in Nigeria; All hospitals, including private ones, must follow ministry guidelines
    - Federal Ministry of Health Intervention and Oversight; Ministry resolves issues in both public and private hospitals; Patients are expected to access antibiotics only through proper prescriptions
    - "Nigeria Food and Drug Control Agency; Ensures quality assurance of all drugs entering or manufactured in Nigeria; Randomly inspects pharmaceutical stores to verify compliance."
    - "Enforcement Challenges for Nigeria Food and Drug Control Agency; Agency sanctions pharmacies selling medications without prescriptions; Limited capacity to enforce regulations."
    - "Regulatory Role of Nigeria Food and Drug Control Agency; Agency conducts random pharmacy inspections to prevent unauthorized medication dispensing; Sanctions violators."
    - "Reaching Doctors through the Nigerian Medical Association; All doctors in Nigeria are members of the Nigerian Medical Association; Periodic meetings serve as a platform for disseminating AMR-related information."
- Interview 10
    - "Case of a non-doctor conducting an appendectomy; Highlights severe issues in medical regulation and oversight."
    - "Efforts to Combat Medical Quackery; Anti-Quackery Committee identifies and apprehends unqualified practitioners; Addresses unauthorized antibiotic prescriptions."

        - "Anti-Quackery Committee works with police and medical officers; Planned visits to NAFDAC and NCDC delayed due to absence."
    - Interview 12
        - "National Association of Resident Doctors (NAD); Presence in All States; Limited Coverage in Remote Areas; Efforts to Improve Healthcare Access."
        - "National Association of Resident Doctors (NAD); Advocacy for Resident Doctors' Welfare; Government Engagement; Public Healthcare Advocacy."
        - "National Association of Resident Doctors (NAD); Strikes and Advocacy; Government Response to Healthcare Issues; Healthcare System Challenges in Nigeria."
        - Medical Council of Nigeria Oversight; Disciplinary Actions Against Healthcare Professionals; Fear-Driven Compliance with Medical Ethics
    - Interview 13
        - "Participation in Stakeholder Meetings; Providing Information for Medical and Dental Practitioners; Guiding Stakeholder Discussions."
        - Role of the Medical and Dental Council of Nigeria; Training of Practitioners; Medical Ethics Oversight; Provision of Data for Registered Practitioners.
        - "Private Healthcare Providers' Associations; Association of Medical and Dental Private Practitioners; Guild of Medical Directors; Representation of Private Health Institutions in AMR Policy."
        - "Involvement of Private Healthcare Associations in AMR Stakeholder Meetings; Cascading AMR Awareness to Private Institutions; Fostering the Right Culture in Healthcare Facilities."
- Collaborative Partnerships
        - Interview 1
            - Nigeria has advanced in AWARE categorization; adapted from literature and incorporated into the essential medicine list; finalized but not yet published;
        - Interview 2
            - Encourage integration of IPC, Drug and Therapeutic Committees, and Pharmacovigilance programs to maximize resources and staff efficiency

    - Established a network of stewardship facilities; support them in accessing funding opportunities through partnerships; involved in CW Palms project
  - Interview 3
    - "Microbiologists have established intervention systems for antimicrobial stewardship at various levels in clinical practice."
  - Interview 4
    - A task force has been established to identify and regulate medical laboratory practitioners in Nigeria.
  - Interview 5
    - WHO supports procurement and supply chain of antibiotics, vaccines, and diagnostics; committee member assisting in guideline implementation and development
    - WHO is a key technical agency in Nigeria's AMR response; provided technical and financial support for developing the National Action Plan over the last two years
    - Private initiatives like Peishan are emerging to revitalize primary healthcare, despite the primary focus on the public sector.
    - WHO is taking initial steps to involve military and other hospitals in AMR stewardship, assessing data and capacity before expanding their role; collaborating with CHAN and other organizations to increase private sector involvement
    - Various international agencies and donors implement AMR programs using different guidelines; goal is to create national guidelines for a unified approach and synchronized implementation
  - Interview 6
    - Collaboration with NCDC to improve drug knowledge at the primary healthcare level, focusing on correct dosing, drug choice, and quality
    - Opportunities exist for collaboration with partner organizations, academia, and the

private sector to review policies and involve key stakeholders in strengthening AMR response
        - Academia and the private sector are being actively involved in discussions to review and strengthen AMR policies; ministry agencies are working on passing these regulations
        - Community pharmacists and patent medicine vendors were involved in previous AMR efforts, with a focus on tracking antibiotic sales and treatments; solid advocacy networks are needed.
    - Interview 8
        - Organizational Collaboration in TB Management; Works with prescribers, doctors, and government to develop TB treatment strategies; Supports the classification of essential medicines
        - Influence of Religion and Community Leaders; Religious beliefs and community leaders impact healthcare decisions; Traditional and social structures influence prescribing practices
        - "WHO Support for AMR Efforts; WHO is ready to support initiatives reducing indiscriminate prescribing; Promotes responsible antimicrobial use."
        - Community and Government Support for Disease Prevention; Organizations and government aid in preventing disease outbreaks
        - Establishing AMR Stewardship Programs; Setting up committees or AMR stewardship programs in private healthcare facilities; Collaboration with WHO can support implementation
    - Interview 9
        - Additional Channels for Reaching Doctors; Information can be disseminated through affiliate associations like the National Association of Resident Doctors; Covers a large network of physicians.
        - "Reaching Private Practitioners; Information can be disseminated through the Association of Private Practitioners in Nigeria; Ensures

communication with doctors who primarily work in private facilities."
        - Ministry of Health as a Communication Channel; Ministry can communicate directly with all doctors in Nigeria through hospital heads; Ensures nationwide awareness of AMR policies and best practices
    - Interview 10
        - "Educational Initiatives on Antimicrobial Stewardship; Committee engages with members through hospital visits, seminars, and awareness programs."
        - "Antimicrobial Stewardship and Anti-Quackery Committees monitor private and public healthcare facilities."
        - "Anti-Quackery Committee and Unqualified Practitioners; Committee inspects hospitals for compliance; Some individuals practice medicine without proper qualifications."
    - Interview 11
        - "PNP's National-Level Collaboration on AMR; Engaged in joint efforts with the Nigerian Medical Association and other medical stakeholders."
        - "Past Partnerships on AMR Initiatives; Collaborated with the Lagos State government on antimicrobial resistance efforts."
        - "PNP's Collaborative Efforts on AMR Prevention; Partnered with the Nigerian Medical Association and Lagos State government; Engaged with other relevant organizations."
        - "Private Sector Partnerships for AMR Prevention; Collaborated with private organizations to advocate for antimicrobial resistance prevention."
        - "PNP's Partnership with Laboratories on AMR; Collaborated with Planar Lancet and other labs."
        - "PNP's Collaboration with Clinical Laboratories; Partnered with multiple clinical labs, including Planar Lancet."

        - "Role of Regulatory Bodies in AMR Control; The Medical and Dental Council of Nigeria plays a crucial role in regulating healthcare institutions; Collaborative efforts between regulatory bodies can improve AMR prevention."
        - "Key Stakeholders in AMR Control; The Nigerian Medical Association is the professional umbrella body for doctors; The Medical and Dental Council of Nigeria plays a key role in AMR oversight."
        - "Private Sector Collaboration with Professional Bodies; The private sector can collaborate with professional bodies like the Nigerian Medical Association; ANPMP is a key actor in these partnerships."
    - Interview 12
        - Community Health Workers Filling Gaps; Improving Rural Healthcare Attractiveness; Need for Social Amenities; Better Remuneration for Rural Healthcare Workers
        - "Media Coverage for Drug Malpractice Awareness; Involving Religious, Traditional, and Political Leaders; Consequences for Malpractice."
        - "Accountability in Healthcare; Consequences for Medical and Pharmaceutical Actions; Importance of Funding Allocation."
    - Interview 13
        - "Leveraging Strategic Framework for AMR Awareness; Sector-Wide Approach (SWAp); Collaboration Between Multiple Stakeholders; Involvement of Ministries of Agriculture and Health."
        - "Impact of Stakeholder Actions on Healthcare Delivery; Leveraging Sector-Wide Approach (SWAp) to Improve Antimicrobial Resistance (AMR)."
        - "Role of External Stakeholders in AMR; Support from Development Partners; Collaboration with Nigerian Government and Relevant Agencies for AMR Awareness and Policy Implementation."

        - "Strengthening Stakeholder Collaboration for AMR; Involvement of Ministries of Agriculture, Health, Education, Environment, and Veterinary Doctors; Unified Approach to AMR."
    - Interview 14 (Focus on Public Sector here)
        - "AMR Stewardship in Private vs. Public Sector; Lack of Organization in Private Sector Stewardship; Support from International Organizations for Government Hospitals."
        - Government-Private Sector Collaboration During COVID-19; Successful Model for Wide-Scale Testing.
        - Government-Private Sector Collaboration in Health; Expanding Reach Through Private Facilities and Laboratories; Ongoing Discussions on Engaging Private Sector in AMR Policy
        - "Essential Medicine List Committee in Nigeria; Review of Essential Medicines for the Handbook; Role of the Ministry of Health in Drug Selection."
        - "Adapting WHO AwaRe Classification to Nigeria's Essential Medicine List; Review and Categorization of Antibiotics; Restricting Overuse of Third-Generation Cephalosporins."
        - "Importance of WHO AwaRe Classification for Nigeria’s Essential Medicine List; Policy Development for Antibiotic Categorization; Implementing AMR Policies in Healthcare."
        - "Grant from Commonwealth Pharmacists Association; UK Fleming Fund Support for AMR Stewardship; Capacity Building for Pharmacists in Low- and Middle-Income Countries."
        - "Initiation of AMR Stewardship Programs in 2 More Hospitals; Success in Implementing Stewardship Programs; Fleming Fund Commonwealth Pharmacist Association Grant."
        - "Common AMR Stewardship Strategies; Prospective Audits and Point Prevalence Surveys; Estimating Patterns of Antimicrobial Use; Consistency in Conducting Point Prevalence Surveys."

Theme #2: Role of Practitioners

- Barriers to effective stewardship
  - Inconsistent Prescribing Practices
    - Inconsistent and empirical prescribing by physicians contributes to antimicrobial resistance and stewardship challenges.
      - Interview 1
        - Diagnostics are required for most hospital patients; empirical treatment is uncommon in the private sector; thorough evaluation benefits both patient and hospital
      - Interview 2
        - Dogmatic prescribing behaviors hinder stewardship implementation; facilities report challenges; mitigation strategies in place with positive feedback.
      - Interview 3
        - "Private sector includes both clinicians and non-clinicians; some health workers run small practices and prescribe based on patient affordability rather than proper protocols." [Interview 3]
      - Interview 4
        - Trial-and-error prescribing leads to antimicrobial resistance; a major issue in healthcare practice
      - Interview 6
        - Lack of coordination in the private sector leads to trial-and-error approaches in antimicrobial treatment; patients' ability to pay influences the strength of antibiotic treatment
      - Interview 8
        - "Impact of Indiscriminate Prescribing; Indiscriminate prescribing increases antimicrobial resistance; Importance of educating healthcare providers on responsible prescribing
        - Antimicrobial Stewardship and Monitoring; Responsible prescribing is part of antimicrobial stewardship; Monitoring usage ensures proper implementation

        - Barriers to Prescribing and Healthcare; Lack of knowledge as a barrier for prescribers and the health sector
        - Need for Evidence-Based Improvement; Observations in-country highlight areas for improvement; Many practitioners may not base decisions on evidence-driven practices
        - Importance of Guidelines and Knowledge; Prescribers need guidance on responsible antimicrobial use; WHO guidelines support proper prescribing.
    - Interview 9
        - "Expected Prescription Protocol; Patients are expected to access antibiotics only through prescriptions from qualified personnel; Both public and private facilities should follow this protocol."
        - Diagnostic Uncertainty; Physicians may sometimes be unsure of the exact diagnosis, affecting prescription decisions; Although not common, this can lead to improper antibiotic use.
        - "Uncertainty in Dosage; Physicians may be unsure of the correct dosage for certain medications; This uncertainty can lead to incorrect prescriptions and potential harm."
        - "Difficulty in Prescribing Antibiotics; Physicians may face challenges in prescribing antibiotics; This can stem from diagnostic uncertainty or lack of proper guidelines."
        - Challenges in Empirical Prescription; Empirical prescribing is common due to delays in diagnostic results; Physicians often prescribe antibiotics without complete information.
        - "Empirical Prescription Based on Symptoms; Physicians prescribe antibiotics based on patient complaints, symptoms, and examination findings; Used when diagnostic testing is delayed."
        - Challenge of Empirical Prescription; Physicians often prescribe antibiotics without laboratory investigations; Empirical prescribing is

necessary when diagnostic results are unavailable
    - "Prescription Documentation Issue; Medical practitioners sometimes prescribe medications without using prescription sheets."
    - "Issue with Prescription Documentation; Lack of prescription sheets leads to informal documentation; Ensure proper documentation for all medical prescriptions to improve tracking."
    - "Proper Prescription Documentation; All prescribed medications should be documented on a prescription sheet; Prevent informal practices like using random pieces of paper."
    - "Verbal Prescription Issue; Some doctors give verbal prescriptions instead of written ones; Practices like verbal prescriptions contribute to antimicrobial resistance."
  - Interview 10
    - "Failure to Follow National Guidelines; Doctors often skip investigations before prescribing antimicrobials; National guidelines from NAFDAC and NCDC exist but are not strictly followed."
    - "Unregulated Access to Antimicrobials; Widespread availability of antibiotics without prescriptions; Lack of proper microbiology investigations before use."
    - "AMR as a Hospital and Societal Issue; Widespread antibiotic abuse leading to resistance; Significant problem in both hospitals and the broader society in Nigeria."
    - Lack of awareness and resistance to change; Long-standing habit of dispensing medications without prescriptions persists.
  - Interview 11
    - "Challenges in SOP Implementation in Private Sector; Limited implementation of SOPs in private institutions; Few private healthcare providers in the region follow proper SOPs."
  - Interview 12

        - "Profit-Driven Unqualified Prescriptions; Inappropriate Antibiotic Use for Symptoms; Lack of Proper Diagnosis Before Medication."
        - "Pharmacists and Medical Workers Acting as Prescribers; Lack of Diagnostic Training; Inappropriate Antibiotic Selection and Timing."
    - Interview 13
        - Role of Private Healthcare Practitioners; Importance of Proper Antibiotic Prescription in Private Sector; Reporting Antibiotic Use to Relevant Authorities.
        - Barriers in Drug Prescription; Debate Over Who Should Be Prescribers; Roles of Doctors, Pharmacists, and Nurses in AMR
    - Interview 14
        - Behavioral Change in Clinical Practitioners; Challenges in Shifting Long-Established Practices; Need for Gradual and Targeted Programs for AMR Stewardship.
        - Challenges in Changing Antibiotic Use Behaviors; Evidence-Based Need for Improved Practices; Mentorship for Private Healthcare Facilities; Gradual Behavioral Change
- Training Limitations and Low Awareness Among Practitioners
    - Interview 6
        - Limited awareness and training capacity are challenges, as only a few people per facility are trained, even within public sector facilities, hindering broader AMR education
        - Financial incentives are a challenge for training participation, as some healthcare workers question the value of attending training when they could be earning more
        - Financial concerns hinder participation in training and meetings, as healthcare workers can earn significantly more from clinical work than attending events; lack of incentives to train staff
    - Interview 8
        - Barriers to Prescribing and Healthcare; Lack of knowledge as a barrier for prescribers and the health sector

- ■ Need for Evidence-Based Improvement; Observations in-country highlight areas for improvement; Many practitioners may not base decisions on evidence-driven practices
- ■ Importance of Guidelines and Knowledge; Prescribers need guidance on responsible antimicrobial use; WHO guidelines support proper prescribing.
- ■ Facility-Level Awareness and Challenges; Many healthcare facilities lack awareness of antimicrobial resistance; Providing education helps address the challenge

- Opportunities for AMR-related activities in the private sector
  - Evidence-Based Prescribing Practices
    - Interview 1
      - "Sending patients on a culture if suspected infection and placing them on broad-spectrum antibiotic"
      - "Resistance concerns; selecting between Augmentin, Cefixime, and Ceftriaxone"
    - Interview 2
      - "When waiting for culture results, place patients on broad-spectrum antibiotics. Will see if it’s sensible to determine treatment options."
      - "Encourage testing before starting antibiotics; ensure patients complete the full prescribed course"
    - Interview 5
      - Some private hospitals have diagnostic facilities and conduct testing, but they are not included in the national AMR surveillance network; testing is done mainly for internal hospital use.
  - Training Resources for Practitioners
    - Ongoing education through standardized modules, hospital seminars, and government support enhances healthcare worker competency in antimicrobial stewardship.
      - Interview 1
        - "Routine trainings required for medical license renewal; must meet minimum score; online options"
        - "Private providers organize scientific lectures to update members on current trends"

- Interview 2
  - Trained healthcare workers to implement antimicrobial stewardship in 52 hospitals; coverage remains limited given Nigeria's size and needs
- Interview 5
  - WHO has developed training modules for AMR stewardship in Africa, aimed at supporting all African countries
  - WHO training modules were piloted in Nigeria, Zambia, and Ghana; awaiting launch before broader implementation; will establish a standardized AMR stewardship framework
- Interview 6
  - Training 120,000 frontline health workers to address essential drugs, commodities, and AMR issues at the primary healthcare level; goal to prevent indiscriminate antimicrobial use
  - Training frontline healthcare workers at the PHC level to understand AMR, correct prescribing practices, and the role of stewardship; addressing misconceptions
  - National and state-level training conducted, with a focus on LG-level cascade to implement antimicrobial stewardship; educating healthcare workers on essential drug use.
  - Private sector was involved in training during the COVID-19 era, but primary healthcare facilities (government-run) are the core focus of the organization's mandate.
- Interview 10
  - "Educational Initiatives on Antimicrobial Stewardship; Committee engages with members through hospital visits, seminars, and awareness programs."
  - Ensuring Compliance with Antibiotic Guidelines; Members are educated on correct antibiotic use; The association enforces adherence to proper guidelines
- Interview 14

            - "Government Support for Private Health Facilities; Providing Training Materials on Diagnostic and Antimicrobial Stewardship."
    - Strategies to enhance private practitioners' engagement in AMR response
        - Scaling Training
            - Interview 2
                - Plan to engage private sector through advocacy tools and training; initial engagement with the Guild of Private Medical Practitioners to gain buy-in
                - "Routine trainings required for medical license renewal; must meet minimum score; online options"
            - Interview 3
                - "Need for more training opportunities; propose identifying private facilities within communities to educate them on proper antimicrobial use."
                - "Training needed at the community level to standardize practices; tertiary institutions implement stewardship, but secondary, primary, and private sectors lack proper stewardship programs."
                - Organized trainings and engaging more microbiologists to spread education could help address antimicrobial resistance challenges.
                - Develop educational materials like leaflets and posters for health facilities; strategically placed to reinforce training and promote guideline adherence
            - Interview 4
                - Strengthen capacity through personnel training and equipment availability to enhance antimicrobial stewardship and diagnostics
                - Strengthen private sector capacity through training, equipment, and affordable lab consumables to enhance Nigeria's AMR response
            - Interview 5
                - AMR guidelines and training modules have been developed but not yet published or launched; final stages of completion to support stewardship implementation

        - Standard treatment guidelines need promotion at the subnational level to reduce antibiotic overuse and misuse; key priority is private sector involvement from planning stages
        - WHO has developed training modules for AMR stewardship in Africa, aimed at supporting all African countries
    - Interview 6
        - Private sector buy-in can be achieved through policy linkage to accreditation and license renewal, but success depends on strong advocacy and consistent engagement
        - Training private sector facilities on antimicrobial stewardship and ensuring understanding is crucial, but challenges like attrition must be considered to maintain consistency.
    - Interview 7
        - Private sector; Clinician's role in improving care and communication
        - Stewardship practices; analyzing the patient's age, drug availability, and affordability
        - Stewardship practices; Looking at clinical experience, performing sensitivity testing
        - Solutions for improving prescribing practices; Need for in-house training, collaboration with physicians and pharmacists
        - Solutions for improving prescribing practices; Awareness of new drugs, benefit from drug representatives, updates on drug effectiveness and combinations.
        - Solutions for improving prescribing practices; Importance of education and retraining for clinicians.
        - Strategies for improving antimicrobial use; Importance of training, limited strategies beyond what is taught
    - Interview 8
        - Training and Awareness for Private Sector; Educate private healthcare providers on proper prescribing; Highlight benefits and risks of antimicrobial use to improve prescribing behavior

        - Educating Private Sector Prescribers; Awareness and education promote responsible prescribing; Key to mitigating antimicrobial resistance’
        - Facility-Level Awareness and Challenges; Many healthcare facilities lack awareness of antimicrobial resistance; Providing education helps address the challenge
        - Private Sector’s Role in AMR Mitigation; Educating private sector health practitioners increases awareness of AMR; Responsible prescribing in private healthcare settings.
        - Role of Workshops and Leadership Engagement; Training workshops for healthcare facility leaders increase awareness; Leadership involvement supports antimicrobial stewardship
        - Private Sector’s Role in AMR Mitigation; Educating private sector health practitioners increases awareness of AMR; Responsible prescribing in private healthcare settings.
        - Role of Education in Overcoming Barriers; Training increases prescribers’ awareness of antimicrobial resistance; Education leads to more informed and responsible prescribing
        - Overcoming Resistance to Change; Experienced prescribers may be reluctant to adopt new practices; Education and WHO guidelines can encourage acceptance.
        - Rapid Dissemination of IPC Messages; Infection prevention and control (IPC) messages reached both healthcare professionals and the public
        - Complementary Strategies for AMR Mitigation; Pilot study complements practitioner training; Combining facility sampling and education strengthens AMR interventions
        - Encouraging Responsible Dispensing Practices; Pharmacies and chemists should inquire about patient history before dispensing antibiotics; Helps curb indiscriminate antibiotic use
    - Interview 9
        - Strategy to Limit Indiscriminate Prescription; Increase the number of qualified health workers

(doctors, pharmacists); Ensures better access to healthcare and reduces misuse.

    - Strategy for Equitable Distribution of Medical Personnel; Ensure equitable distribution of medical staff across Nigeria; Mandate trained pharmacists in every hospital
    - Strategy to Improve Healthcare Access; Increase the number of trained health workers, doctors, and pharmacists; Addresses the issue of accessibility and availability.
    - Addressing AMR requires strategies at multiple levels; Solutions include employing more personnel, training, and enforcing legal actions for non-compliance
- Interview 12
    - "Community Health Workers; Education on Antibiotic Use; Referral Systems in Rural Areas; Commonality of Antibiotic Resistance and Misuse."
    - Expanding Healthcare Workforce; Benefits for Public and Private Sectors; Strengthening Rural Healthcare; Increasing Health Awareness Opportunities
- Interview 13
    - "Strategy for AMR Advocacy; Incorporating AMR Education into Undergraduate Curriculum; Training Future Healthcare Professionals to Combat AMR."
    - "Capacity Building through Continuous Professional Development (CPD); Mandatory Credit Units for Medical Practitioners; Ongoing Education for Healthcare Professionals."
    - "Integrating AMR into CPD Activities; Enhancing Education on AMR for Doctors and Healthcare Providers."
    - "Including Private Providers in AMR Stakeholder Meetings; Motivating Through Recognition; Enhancing AMR Awareness and Implementation in Private Health Institutions."
- Interview 14
    - Mandatory Refresher Courses for Clinical Personnel; Regulatory Requirements for Annual

Practice Licenses; Enhancing Private Facility Standards Through Continuous Training.

- Increasing AMR Awareness
    - Awareness strategies are essential to reduce antibiotic misuse, improve AMR understanding, and strengthen antimicrobial stewardship efforts.
        - Interview 1
            - "Government intervention needed for awareness; use social media, radio, TV, and religious houses to inform the population"
            - "Counsel patients against antibiotic abuse; explain risk of tolerance; lack of awareness is a key issue"
            - "Discourage unnecessary antibiotic use; emphasize need for proper investigation before starting activity"
            - "Encourage testing before starting antibiotics; ensure patients complete the full prescribed course"
            - "Building awareness; Information, Education, and Communication (IEC) strategies used"
            - Private practitioners' role in explaining antibiotics, not forcing
        - Interview 2
            - "Importance of explaining to patients the source of antimicrobial resistance; illness"
            - "Importance of Encouraging patients to use antibiotics properly; avoid leftover antibiotics"
            - Encourage testing before starting antibiotics; ensure patients complete the full prescribed course
        - Interview 3
            - Social media, radio, and electronic media are key for AMR awareness; jingles on radio stations can remind the public about antimicrobial resistance and stewardship
            - Develop educational materials like leaflets and posters for health facilities; strategically placed to reinforce training and promote guideline adherence
        - Interview 4

        - Awareness is not low; increasing awareness in major institutions can further strengthen AMR prevention efforts
        - Leverage traditional, religious, and political leaders to drive AMR awareness and compliance
    - Interview 5
        - Standard treatment guidelines need promotion at the subnational level to reduce antibiotic overuse and misuse; key priority is private sector involvement from planning stages
    - Interview 6
        - Engagement is the first step in involving the private sector in AMR efforts, but the vastness of the sector and the number of people involved make it challenging to implement effective AMR programs.
        - Increased awareness of antimicrobial resistance is driving a focus on policy regulation, prompting active engagement and questions at meetings about the ongoing policy efforts
        - Enlightenment and awareness are key; integrating antimicrobial resistance into in-service training for all training institutes is important to raise awareness
    - Interview 7
        - Preventing antibiotic abuse; Educating patients on correct usage, reviewing drug use during follow-up, challenges with patient literacy and follow-up
        - Preventing antibiotic abuse; Educating and improving communication with patients
        - Key strategies; Clinical experience, patient interaction
        - Awareness and knowledge in private sector; Monthly lectures from drug companies, reading materials
    - Interview 8
        - Follow-Up and Treatment Review; Patients should return to the prescriber if there is no improvement; Ensures appropriate adjustments to treatment

- Prescription Adherence and Duration; Prescriptions are given based on disease and treatment period; Patients are expected to follow prescribed duration for effectiveness
- Spreading Awareness Through Healthcare Professionals; Educated healthcare professionals can cascade AMR awareness to patients and the community.
- Private Sector’s Role in AMR Mitigation; Educating private sector health practitioners increases awareness of AMR; Responsible prescribing in private healthcare settings.
- Role of Community-Based Organizations; Community organizations are effective in raising awareness; Similar approaches can be used to educate on antimicrobial resistance
- AMR in Agriculture and Community Awareness; Antimicrobial resistance affects both humans and animals; Farmers need education on the risks of indiscriminate antimicrobial use.
- "Expanding Awareness Strategies; Awareness should extend beyond word of mouth; Utilize phones, TV, and radio to reach a wider audience in the community."
- "Use radio, TV, and social media to disseminate AMR information; Expanding outreach through various media enhances public engagement."
- "Targeted AMR Awareness Programs; Separate initiatives for practitioners and community members; Organize practitioner-focused training and community outreach."
- "Traditional Awareness Methods; Pamphlets and leaflets have been used to convey health information; Printed materials can support AMR awareness efforts."
- "Community Awareness Initiatives; Monthly jingles can reinforce AMR awareness; Workshops and public drives can enhance community engagement."
- "Impact of Clear Public Health Messaging; Multi-platform communication improves adherence."

        - "Effective Media Campaigns in Outbreaks; Jingles and media advisories were effective during past outbreaks; Public health messaging influences behavior by emphasizing AMR risks."
        - Lessons from Ebola Outbreak; Extensive public awareness efforts were used during the Ebola outbreak
        - Rapid Dissemination of IPC Messages; Infection prevention and control (IPC) messages reached both healthcare professionals and the public
        - Encouraging Responsible Dispensing Practices; Pharmacies and chemists should inquire about patient history before dispensing antibiotics; Helps curb indiscriminate antibiotic use
    - Interview 9
        - "AMR Awareness Strategy; Focus on sustained and widespread awareness of antibiotic abuse dangers; Aims to reduce misuse and improve responsible prescribing."
        - "AMR Awareness Strategy; Mass and sustained awareness through various media (electronic, print, social); Educate the public on the dangers of self-prescription and antibiotic misuse."
        - Addressing AMR requires strategies at multiple levels; Solutions include employing more personnel, training, and enforcing legal actions for non-compliance
    - Interview 10
        - Training and Media Engagement for AMR Awareness; Organize regular antimicrobial stewardship training; Use mass media for public education beyond hospitals
    - Interview 11
        - Focus on Advocacy for AMR Prevention; Belief in the power of advocacy to improve antimicrobial resistance prevention; Strong emphasis on public health initiatives.
        - Advocacy as a Key Strategy for AMR; Strong belief in using advocacy to improve public health and quality of life; Prevention emphasized as the most effective approach.
    - Interview 12

        - "Strengthening Primary Healthcare; Community Engagement in Healthcare; Role of Media and Traditional Leaders in Health Education."
        - Role of Healthcare Workers in Public Education; Community Engagement to Reduce Antibiotic Resistance; Improving Quality of Life in Rural Areas
        - Community Leadership in Healthcare Advocacy; Engaging Religious, Village, and Political Leaders; Ongoing Efforts to Improve Healthcare; Hope for Policy Change
        - "Media Coverage for Drug Malpractice Awareness; Involving Religious, Traditional, and Political Leaders; Consequences for Malpractice."
        - Expanding Healthcare Workforce; Benefits for Public and Private Sectors; Strengthening Rural Healthcare; Increasing Health Awareness Opportunities
        - "Community Engagement for Antibiotic Stewardship; Role of Traditional and Religious Leaders; Addressing Widespread Drug Abuse in Nigeria."
        - "Role of Guidelines in Proper Medication Use; Community Leadership in Curbing Drug Abuse."
    - Interview 14
        - "Education for Policymakers on AMR; Raising Awareness Among Policymakers about the Importance of AMR, Surveillance, and Stewardship."
- Promoting Protocol-Driven Prescribing
    - Interview 1
        - "Encourage testing before starting antibiotics; ensure patients complete the full prescribed course"
    - Interview 4
        - Primary care plays a crucial role in AMR; many referrals happen after trial-and-error treatments; proper antibiotic use at this level can reduce unnecessary referrals.

        - Strengthen quality control in medical laboratory science through evolving regulatory bodies to improve diagnostics.
    - Interview 6
        - Focusing on primary prevention is key. If we prevent infections early, we reduce the need for antibiotics, thus limiting resistance. Strengthening infection prevention efforts will improve long-term AMR outcomes
    - Interview 10
        - "Hospital Protocols for Antibiotic Use; Preliminary investigations conducted before administration; Empirical antibiotic use followed by culture and sensitivity-guided prescription."
        - "Standard medical centers follow proper protocols; Issues persist in hinterlands and unregulated pharmacies."
        - "Emphasize culture and sensitivity testing; Prevent pharmaceutical companies from indiscriminately supplying antibiotics."
        - "Improving Diagnostic Accuracy and Regulating Drug Supply; Emphasize culture and sensitivity testing; Prevent pharmaceutical companies from indiscriminately supplying antibiotics."
    - Interview 11
        - "Importance of SOPs in Healthcare; Standard Operating Procedures (SOPs) are crucial in clinical practice; Advocate for SOPs in all private healthcare institutions as a fundamental guideline."
        - "SOPs as Clinical Guidance; Implement SOPs in private sectors and secondary healthcare institutions; SOPs should guide clinical practices across various healthcare settings."
        - "SOPs to Guide Clinical Practices and Combat AMR; SOPs in private and primary healthcare institutions to ensure proper practices; Helps reduce antimicrobial resistance."
        - "Implementing SOPs for AMR Control; Focus on establishing Standard Operating Procedures

                (SOPs) in clinical settings; Advocate for the adoption of SOPs or Manuals."
            - "Global Standard SOPs for AMR Prevention; Establishment of globally accepted guidelines for private healthcare practitioners; SOPs will improve antimicrobial resistance management."
            - "Key Strategies for AMR Prevention; Advocate for effective SOPs in collaboration with the State government; Focus on securing funding through collaborative proposals."
        - Interview 12
            - "Public Awareness on Antibiotic Use; Importance of Seeing a Doctor Before Using Antibiotics; Strengthening Primary Healthcare for Systemic Improvement."

Theme #3: Role of Government/Health Systems

- Burden of AMR
    - Interview 1
        - "Low commonality of AMR in practice; it still exists"
    - Interview 2
        - Global momentum growing to address AMR before it erupts as a pandemic
    - Interview 3
        - "AMR is a critical issue in Nigeria; high resistance to several antimicrobials due to widespread usage."
        - "Patients receive incomplete antibiotic courses; contributes to high AMR cases in healthcare facilities."
    - Interview 4
        - "AMR complicates treatment of otherwise simple infections; leads to patient complications and higher government healthcare costs."
        - Containing AMR in Nigeria will have a significant positive impact on Africa's overall health security.
    - Interview 5
        - AMR is a major issue in Africa, especially in low- and middle-income countries; often called a silent pandemic; data remains poorly documented.
        - Stewardship is not a primary focus within Peishan; Nigeria faces major healthcare challenges, including

emergencies, service delivery, accessibility issues, and medication shortages
        - Antibiotic overuse is highest at the community level; lack of healthcare facilities in rural areas drives demand; unregulated vendors and unlicensed pharmacists supply antibiotics
        - People seek nearby care, often from untrained providers; rely on religious practitioners, quacks, and unlicensed individuals instead of trained healthcare professionals
    - Interview 6
        - Exact AMR prevalence figures in Nigeria are unavailable, but it is known to be high; antimicrobials should only be prescribed by certified personnel
    - Interview 11
        - "High Prevalence of AMR in Nigeria; Research indicates over 64,000 deaths in 2019 were directly linked to antimicrobial resistance."
        - "AMR-Associated Morbidity and Mortality; Studies estimate over 260,000 conditions or deaths linked to antimicrobial resistance."
        - "Highest AMR Rates in Nigeria; Nigeria has the highest antimicrobial resistance in humans."
- System-level barriers
    - Infrastructure and Resource Constraints
        - Interview 1
            - "Issue of availability of antibiotics"
        - Interview 2
            - "Resource constraints challenge full implementation; prioritizing public facilities for easier access and monitoring; plan in place to include private sector later."
        - Interview 5
            - Antibiotic access is a challenge; some are unavailable, while others are overused; both access and excess contribute to AMR in Nigeria
            - Registration, procurement, and distribution of antibiotics need stronger regulation; current processes are not properly regulated.
        - Interview 7
            - Barriers to good prescribing practices; Availability of drugs

        - Interview 9
            - Medication Availability Challenge; Many hospitals in Nigeria lack medications in their stores; Patients are required to purchase medications from pharmacy stores
            - "Issues with Medication Availability; Many hospitals lack necessary medications, despite having drug-revolving funds; Inefficient management of resources leads to supply shortages."
        - Interview 10
            - "Systemic Gaps in AMR Control; Limited laboratory capacity to identify infections; Gaps in access to effective antimicrobials and funding; Lack of training for skilled personnel."
        - Interview 11
            - "Improving Private Healthcare Facilities for AMR; Many private facilities lack proper infrastructure for microbial testing; Need for appropriate cultural mediums, SOPs, and guidelines."
            - "Gaps in AMR Control; Issues with laboratory capacity to identify infections; Gaps in access to effective antimicrobials, funding, and planning; These gaps hinder the success of AMR strategies."
        - Interview 12
            - "Weak Primary and Secondary Healthcare Systems; Overburdened Tertiary Healthcare; Healthcare System Dysfunction in Nigeria."
            - "Strengthening Primary Healthcare; Reducing Healthcare System Burden; Public Understanding of Antibiotics."
            - Private Healthcare Preference for Profitable Areas; Financial Challenges in Rural Healthcare; Irregular Payment for Services; Barriers to Healthcare Investment in Remote Regions
- Workforce Shortages
    - Lack of healthcare personnel strains antimicrobial stewardship, leading to improper prescribing and AMR spread
        - Interview 1

        - "Challenge of laboratory scientists traveling for better work conditions, patients must travel further for lab work"
        - Challenges in human capacity and stewardship oversight; high healthcare worker attrition due to migration for better opportunities; workforce shortage strain."
    - Interview 2
        - Healthcare worker shortages due to migration; advocacy for more employment, but many leave soon after for better-paying jobs; challenge beyond local intervention.
    - Interview 3
        - "Shortage of trained personnel to educate communities on proper antibiotic use; critical for those handling medications and patient care."
        - "Major challenge is manpower shortage; few microbiologists available to educate physicians and health workers on antimicrobial resistance."
    - Interview 4
        - Ensure properly trained personnel conduct laboratory services; brain drain remains a challenge, but capacity building can help strengthen the workforce
    - Interview 5
        - Hospitals face significant human resource shortages; integrated approach needed to optimize resources at the hospital level
    - Interview 6
        - "Human resource shortages impact proper antimicrobial prescription; doctor-to-patient and nurse-to-patient ratios are low; task-shifting and task-sharing policies."
        - "Organization lacks dedicated staff for antimicrobial stewardship at the primary healthcare level; raised this concern with the Nigeria Center for Disease Control (NCDC)."
        - Primary healthcare faces challenges such as human resource shortages, commodity stock-

outs, and inadequate financing, driving people to seek care in the private sector.
        - "Lack of sufficient human resources in private sector facilities is a key limitation, making it difficult to assign dedicated staff for AMR reporting and management duties."
        - "Staff in private sector facilities often function in dual roles, such as nurses doubling as pharmacists, due to limited human resources, complicating the implementation of AMR programs."
        - "Challenges in private sector facilities include lack of dedicated personnel; nurses may take on multiple roles, such as acting as physicians or pharmacists, due to staffing shortages."
    - Interview 7
        - Staff shortages in private sector facilities make it difficult to release employees for training, as the need for workforce continuity creates additional strain on already limited human resources
        - Staff turnover in the private sector presents a challenge for training continuity, as trained workers may leave soon after, requiring repeated training efforts to maintain trained personnel
        - High staff attrition due to better opportunities elsewhere is a challenge for both public and private sectors, affecting training continuity; time constraints in the private sector also hinder participation
        - "Manpower challenges in private sector facilities mirror those in public facilities, with concerns about workload, payment, and feasibility of assigning dedicated AMR personnel."
    - Interview 9
        - Healthcare Access Challenge; Poor doctor-patient ratio in Nigeria; Leads to non-doctors prescribing medication due to limited access to doctors

        - Factors Affecting Healthcare Access; Limited availability and high cost of healthcare; Uneven distribution of doctors, with more concentrated in urban areas
        - Rural Healthcare Challenges; Rural areas have a shortage of doctors; Patients turn to community health workers or unqualified individuals for prescriptions; Informal prescribing remains a challenge
        - "Lack of sufficient medical personnel and uneven distribution across Nigeria; Some communities have very few healthcare workers, leading to limited access for large populations."
        - "Healthcare Access Challenge; Limited medical personnel in some communities prevents proper prescription access; The issue requires multi-level solutions."
    - Interview 10
        - "Doctors in private practice handle multiple roles; Manpower shortages lead to reliance on empirical antimicrobial treatments."
        - Workforce and Distribution Challenges in AMR Control; Shortage of skilled personnel to support hospital management; Poor distribution of antimicrobial medications
    - Interview 12
        - "Limited Healthcare Access; Pharmacies as Primary Healthcare Providers; Delayed Hospital Visits Due to Distance; Common Healthcare-Seeking Behavior in Rural Areas."
        - "Limited Healthcare Access in Nigeria; Economic Impact on Healthcare System; Medical Brain Drain; Increased Workload on Remaining Healthcare Professionals."
        - "Rural Healthcare Challenges; Doctor Shortage in Villages; Underutilized General Hospitals."
        - "Community Health Workers Filling Gaps; Improving Rural Healthcare Attractiveness; Need for Social Amenities; Better Remuneration for Rural Healthcare Workers."
        - "Government Intervention in Healthcare; Strengthening Primary Healthcare; Urban-Rural

        - Healthcare Disparities; Doctor Shortage in Rural Areas."
        - "Healthcare Workforce Shortage; Medical Brain Drain in Nigeria; Limited Healthcare Access in Rural Areas; Impact on NAD Coverage."
        - Private Healthcare in Rural Areas; Limited Private Investment in Villages; Profit-Driven Nature of Private Healthcare; Urban Concentration of Private Hospitals
    - Interview 13
        - "Human Resource Shortages in Healthcare."
        - "Staffing Issues in Primary Healthcare Centers; Predominance of Community Health and Environmental Health Practitioners; Lack of Incentives for Health Workers in Rural Areas."
    - Interview 14
        - "Manpower Challenges in the Healthcare Sector; Greater Impact on Public Hospitals; Opportunity to Leverage Private Health Facilities for Training and Capacity Building."
- Limited Leadership and Government Coordination
    - Lack of government involvement and leadership weakens AMR policies, regulation, and implementation efforts.
        - Interview 3
            - Financial constraints lead patients to buy incomplete treatments; health insurance could ensure full-course medication access; government has a major role in addressing affordability.
        - Interview 4
            - The Nigerian Constitution limits federal enforcement over primary healthcare; federal efforts focus on capacity building and coordination, but direct supervision is limited.
        - Interview 5
            - Limited national and state-level involvement in the MRT program; some sub-national facilities participate, but regulation and capacity building remain centralized
            - States need to actively engage in AMR efforts; private sector involvement is lacking; first step

is an assessment to determine how to integrate them effectively.

        - AMR partners work directly with states and communities, not at the national level; national technical working group for stewardship exists, but private sector involvement remains limited

    - Interview 10
        - "Coordination issues and diagnostic uncertainty hinder effective antimicrobial stewardship."
    - Interview 11
        - "Government and Collaboration Challenges in AMR; Lack of strong government support and will can hinder progress; Effective collaboration with stakeholders is essential."
    - Interview 12
        - Lack of Government Presence in Rural Healthcare; Need for Alternative Healthcare Solutions; Strengthening Public Healthcare as a Solution; Limited Private Investment
        - Private Healthcare in Rural Areas; Limited Private Investment in Villages; Profit-Driven Nature of Private Healthcare; Urban Concentration of Private Hospitals
        - "Barriers to Antimicrobial Intervention; Government Policy on Drug Use; Regulatory Challenges in Clinic Management."
    - Interview 13
        - "Governance and Leadership Barriers; Lack of Awareness of AMR Policies in Nigeria; Limited Implementation of AMR Frameworks."
        - "Government Responsibility in Policy Enforcement; Involvement of All Stakeholders in AMR Implementation; Ensuring Effective AMR Policy Follow-Through."
        - "Importance of Political Will in AMR Efforts; Leadership as Key to AMR Success; Challenges in Developing Countries' Attention to AMR."
        - "Prioritizing AMR in Healthcare; Leadership Responsibility in Addressing AMR; Overcoming Competing Health Priorities in Developing Countries."

- Quality and Safety Challenges

- Low-quality drugs and inadequate care undermine antimicrobial stewardship efforts and contribute to AMR.
    - Interview 3
        - "Haphazard prescription patterns in Nigeria; patients obtain antibiotics from small shops and patent medicine stores without trained pharmacists."
        - "Some private facilities are run by non-clinicians; profit often prioritized over antimicrobial stewardship; patients receive partial antibiotic courses based on what they can afford."
    - Interview 5
        - Regulations on antibiotic access exist but are not enforced; free access at the community level, with vendors selling drugs on the street.
        - Antibiotic overuse is highest at the community level; lack of healthcare facilities in rural areas drives demand; unregulated vendors and unlicensed pharmacists supply antibiotics
        - People seek nearby care, often from untrained providers; rely on religious practitioners, quacks, and unlicensed individuals instead of trained healthcare professionals
        - Capacity and antibiotic quality are major issues in Nigeria; falsified and substandard medicines circulate despite regulations and registration; stronger enforcement needed
    - Interview 6
        - Easy over-the-counter access to antibiotics without a prescription is a major driver of AMR in Nigeria; antibiotics can be purchased freely at medicine shops and from vendors
        - Unregulated access to antibiotics contributes to high AMR in Nigeria; street vendors and medicine shops freely distribute antimicrobials without restrictions
        - Private sector facilities are often not regulated to report antimicrobial procurement and usage; lack of tracking in the supply chain and use for specific conditions contributes to AMR.

        - Private sector challenges include procuring cheaper drugs, sometimes at the expense of quality, while public sector facilities have more centralized procurement policies
    - Interview 8
        - Need for Prescription Accountability; Monitoring prescriptions helps track medication use and prevent misuse; Addressing over-the-counter antibiotic sales without regulation
        - Regulating Over-the-Counter Antibiotic Sales; Implementing controls on what medications can be bought without prescriptions; Helps reduce indiscriminate antibiotic use
    - Interview 9
        - "Factors Contributing to AMR; Use of low-quality antibiotics or alterations in their composition."
        - "Factors Contributing to AMR; Circulation of low-quality antibiotics; Reduced potency leads to bacterial resistance."
        - AMR and Drug Quality in Developing Countries; Low-quality antibiotics are more common in developing countries; Limited access to high-quality antibiotics contributes to resistance
        - "Impact of Low-Quality Antibiotics on AMR; Patients may develop resistance even when following prescriptions; Reduced potency of low-quality antibiotics contributes to treatment failure.
        - Risk of Adulterated Drugs; Some pharmaceutical stores dispense adulterated medications; Increases risk to patients' health and contributes to AMR.
        - Challenges in Access to Healthcare; Non-doctors prescribing medication without authorized prescriptions; Patients often receive medications on informal prescriptions
        - Factors Affecting Healthcare Access; Limited availability and high cost of healthcare; Uneven distribution of doctors, with more concentrated in urban areas

        - Medication Availability Challenge; Many hospitals in Nigeria lack medications in their stores; Patients are required to purchase medications from pharmacy stores
    - Interview 10
        - "Unregulated Access to Antimicrobials; Widespread availability of antibiotics without prescriptions; Lack of proper microbiology investigations before use."
        - "AMR as a Hospital and Societal Issue; Widespread antibiotic abuse leading to resistance; Significant problem in both hospitals and the broader society in Nigeria."
        - "Role of the Private Sector in AMR; Significant issues stem from pharmaceutical facilities and chemists; Not solely blaming private medical facilities."
        - "Pharmacies and chemists sell medications without prescriptions; Contributes to antimicrobial misuse and resistance."
        - "Pharmacist Selling Antibiotics Without Prescription; Personal experience witnessing unregulated antibiotic sales; Pharmacist prioritized sales over proper antimicrobial use."
        - "Pharmacist sold medication without a prescription; Incident reported to authorities; Highlights enforcement challenges in private pharmaceutical establishments."
        - "Private medical facilities are association members; Can only advise them on antimicrobial stewardship; Lack of enforcement authority over their practices."
        - "Pharmacists Undermining Doctor Prescriptions; Pharmacists or chemists recommend stronger antibiotics over doctor-prescribed treatments; Contributes to inappropriate antibiotic use."
    - Interview 11
        - Contributing Factors to Antimicrobial Resistance; Poor clinical care, weak regulations, and lack of microbial surveillance; Inappropriate antibiotic use is the most common driver

        - "Patients purchase drugs from chemists due to financial constraints; Lack of access to proper healthcare, including diagnostic tests, contributes to improper use of antibiotics."
        - "Patient Challenges in AMR; Ignorance and poverty lead patients to seek improper care; Financial constraints cause reliance on chemists instead of proper healthcare services."
    - Interview 12
        - Antimicrobial Resistance (AMR); Lack of Regulation on Antibiotic Sales; Self-Medication; Incorrect Antibiotic Use; Delayed Hospital Visits.
        - Private Healthcare Regulation; Government Oversight in Cities; Lack of Regulation in Rural Areas; Quality Control Challenges in Interior Regions
        - Financial Motives in Private Healthcare; Presence of Unethical Practitioners; Regulatory Oversight and Enforcement
        - Medical Ethics in Diagnosis and Referral; Challenges in Proper Patient Referral; Financial Pressures in Healthcare Facilities; Overprescription Due to Operational Costs.
        - Unregulated Healthcare in Villages; Unqualified Individuals Running Clinics; Lack of Medical Knowledge; Challenges in Identifying Unlicensed Practitioners
        - Overprescription Instead of Referral; Delayed Referrals for Severe Infections; Risks of Late-Stage Hospital Transfers; Reporting Cases to Authorities
        - "Counterfeit Drugs as the Primary Barrier; Widespread Fake Medications; Impact on Antimicrobial Treatment Effectiveness."
        - "Prevalence of Fake Drugs; Antibiotic Ineffectiveness Due to Counterfeits; Challenges in Effective Treatment."
        - "Counterfeit Drugs Complicating Treatment; Sensitivity Testing (MCS) Ineffectiveness; Escalation to Stronger Antibiotics."
        - "Counterfeit Antibiotics in Supply Chain; Treatment Failure Due to Fake Medications;

Major Barrier to Effective Antimicrobial Therapy."

- "Suboptimal Active Ingredients in Antibiotics; Contribution to Antimicrobial Resistance; Widespread Issue in Cities and Villages."
- Inequities in Drug Regulation; Favoritism in Pharmaceutical Oversight; Impact of Suboptimal vs. Genuine Antibiotics on Implementation
- Regulation of Drug Production; Penalties for Importing Substandard Medications; Life Sentences for Counterfeit Drug Manufacturers; Reducing Drug Abuse and Antibiotic Resistance
- "Unqualified Practitioners Prescribing Medications; Pharmacists Acting as Diagnosticians; Presence of Quack Prescribers in Cities and Villages."
- "Misdiagnosis by Unqualified Prescribers; One-Size-Fits-All Medication Approach; Lack of Understanding of Disease Variability."
- "Pharmacists and Medical Workers Acting as Prescribers; Lack of Diagnostic Training; Inappropriate Antibiotic Selection and Timing."

- Interview 13
  - "Lack of Prescription Regulation in Developing Countries; Easy Access to Antibiotics Without Prescription; Contrast with Regulated Antibiotic Use in Developed Countries."
- Interview 14
  - Challenges with Proper Investigations in Private Sector; Lack of Routine Cultures for Antibiotic Selection; Cost Considerations Impacting Clinical Decisions.

- Weak AMR Surveillance Systems
  - Limited data and surveillance prevent tracking antibiotic use, resistance patterns, and effectiveness, making it harder to regulate prescribing, detect AMR trends, and implement targeted stewardship interventions.
    - Intervirew 5
      - Limited data on antibiotic use in the private sector, but a significant portion of antimicrobials are used there

        - At least 70% of Nigerians prefer private healthcare, where most antimicrobials are consumed; need stronger private sector involvement in stewardship and data use
        - Some tertiary and secondary private hospitals conduct testing, but many do not fully engage in antimicrobial stewardship
        - National Action Plan 2.0 includes monitoring antibiotic consumption, but surveillance has not started; studies conducted in public hospitals, but private sector data is lacking
        - AWARE classification is being promoted but lacks regular analysis and data compilation; uncertainty about facility participation and activities due to data gaps.
    - Interview 6
        - Exact AMR prevalence figures in Nigeria are unavailable, but it is known to be high; antimicrobials should only be prescribed by certified personnel
        - Data on healthcare services, such as patient numbers, is available from the public sector, but private sector data is lacking, despite its significant presence
        - Non-reporting by the private sector is a key barrier; primary healthcare facilities are required to report monthly data, but private sector data is often not included.
        - Standardized reporting and information flow are key barriers; private practitioners should report to local government authorities, which then report to the state and federal levels
        - Tracking the source and usage of antimicrobial commodities is a challenge; private facilities may purchase antimicrobials without proper tracking of usage and distribution
        - Private sector facilities are often not regulated to report antimicrobial procurement and usage; lack of tracking in the supply chain and use for specific conditions contributes to AMR.
        - Out-of-pocket payments create tracking barriers; lack of records for privately paid

        treatments and reliance on the National Insurance Scheme for some patients hinder data collection
        - Tracking is easier with HMOs, but out-of-pocket payments create barriers to monitoring antimicrobial use and treatment outcomes, making it difficult to track data
    - Interview 9
        - "Issue with Prescription Documentation; Lack of prescription sheets leads to informal documentation; Ensure proper documentation for all medical prescriptions to improve tracking."
    - Interview 11
        - "Challenges in AMR Data and Strategy; Difficulty in obtaining reliable statistics and data; Past collaborative efforts have been made despite these challenges."
    - Interview 14
        - Challenges in Private Sector Antibiotic Treatment; Overuse of Presumptive Antibiotics; Lack of Local Data on Resistance Patterns; Limited Lab Capacity
        - "Monitoring of Antibiotic Use in Nigeria; Role of Government Agencies in AMR Monitoring; Gaps in Effective Monitoring and Use of Indicators."
        - "Monitoring Antibiotic Use in Hospitals; Lack of Systematic Monitoring Across Facilities; Increasing Adoption of Point Prevalence Surveys for Antibiotic Use."

- Inadequate Enforcement
    - Weak enforcement allows unregulated antibiotic use, improper prescribing, and non-compliance, accelerating antimicrobial resistance.
        - Interview 5
            - Limited enforcement of antimicrobial prescription laws; involve police and legal teams in regulation; assess existing laws to ensure proper use of Watch category antibiotics
            - Laws governing antimicrobial prescription and dispensing exist but are not strictly enforced, leading to widespread non-compliance

        - Challenges in antimicrobial stewardship include legislative gaps; laws exist for prescription and dispensing, but enforcement is weak.
        - Over-the-counter antibiotic sales and community-level dispensing contribute to misuse; need stricter regulations to control antibiotic distribution; pharmacists currently lack oversight
        - Registration, procurement, and distribution of antibiotics need stronger regulation; current processes are not properly regulated.
    - Interview 6
        - Unregulated access to antibiotics contributes to high AMR in Nigeria; street vendors and medicine shops freely distribute antimicrobials without restrictions
        - Easy over-the-counter access to antibiotics without a prescription is a major driver of AMR in Nigeria; antibiotics can be purchased freely at medicine shops and from vendors.
        - Lack of policies and regulation in the private sector contributes to AMR and other healthcare challenges.
        - Bringing private facilities into AMR stewardship efforts may be challenging due to concerns about regulation and oversight, but starting from somewhere is crucial
    - Interview 8
        - Regulatory Differences and Access; In Nigeria and Africa, antimicrobial agents are easily available over the counter; In developed countries like the UK and US, access is controlled.
        - Unregulated Access and Resistance; In Nigeria and Africa, antimicrobials are available both by prescription and over the counter; Indiscriminate use contributes to increasing AMR.
        - Need for Prescription Accountability; Monitoring prescriptions helps track medication use and prevent misuse; Addressing over-the-counter antibiotic sales without regulation

        - Regulating Over-the-Counter Antibiotic Sales; Implementing controls on what medications can be bought without prescriptions; Helps reduce indiscriminate antibiotic use
    - Interview 10
        - "Failure to Follow National Guidelines; Doctors often skip investigations before prescribing antimicrobials; National guidelines from NAFDAC and NCDC exist but are not strictly followed."
        - "Issues reported to a regulatory organization; Lack of consistent follow-through on enforcement."
        - "Lack of awareness and weak government regulation; Key challenges in addressing antimicrobial resistance."
        - "Poor regulatory oversight allows misconduct; Lack of proper deterrents leads to continued malpractice."
        - "Government intervention and legislation needed; Proper regulation can improve the system."
        - "Regulations exist but are not enforced effectively."
        - "Federal Ministry of Health must enforce regulations; Street vendors openly sell antibiotics without prescriptions."
        - "Authorities must take action against illegal drug sales; Doctors lack prosecuting power and serve only as guides."
        - "Systemic Lack of Regulation Enabling AMR Practices; Issue stems from weak regulations rather than cultural barriers; Individuals exploit regulatory gaps for financial gains."
        - "Challenges in AMR Control; Weak regulatory mechanisms; Gaps in access to effective antimicrobial agents; Issues in antimicrobial regulation."
    - Interview 11
        - Contributing Factors to Antimicrobial Resistance; Poor clinical care, weak regulations,

            and lack of microbial surveillance; Inappropriate antibiotic use is the most common driver
        - Interview 12
            - "Private Healthcare Sector in Nigeria; National and State-Level Private Practitioner Bodies; Efficiency in Private Healthcare; Presence of Unregulated Hospitals
            - Regulation of Drug Production; Penalties for Importing Substandard Medications; Life Sentences for Counterfeit Drug Manufacturers; Reducing Drug Abuse and Antibiotic Resistance
            - "Unqualified Practitioners Prescribing Medications; Pharmacists Acting as Diagnosticians; Presence of Quack Prescribers in Cities and Villages."
        - Interview 13
            - "Comparison Between Canada and Nigeria; Pharmacy Regulation in Developed vs. Developing Countries; Required Prescription and Consultation in Canada vs. Easy Over-the-Counter Access in Nigeria."
            - "Opportunity for Policy Implementation; Lack of Will to Enforce Existing AMR Policies; Importance of Political Will in Tackling AMR."
            - "Government Responsibility in Policy Enforcement; Involvement of All Stakeholders in AMR Implementation; Ensuring Effective AMR Policy Follow-Through."
- Opportunities
    - Nation-Wide Government Commitments
        - Interview 2
            - "Government role in providing vaccines to reduce antibiotic need"
        - Interview 4
            - Patients seek cheaper healthcare options, often compromising proper treatment; health insurance coverage is still developing; government working on solutions.
        - Interview 6
            - Achieving universal healthcare coverage requires broad stakeholder engagement; international interest and support for AMR

efforts provide opportunities for improved regulations

- Interview 6
  - Immunization plays a crucial role in preventing infections but requires wider availability
  - Vaccines play a crucial role in preventing infections. By focusing on vaccines, especially in high-risk areas, and considering seasonality, we can reduce the need for antibiotic use and prevent antimicrobial resistance
- Interview 8
  - Nigeria collaborates with the government to address TB drug resistance; Development of a ranked medication system
  - Government Collaboration for Structured Prescribing; Work with the government to develop prescribing strategies.
- Interview 9
  - Government Initiatives on Rational Prescription; Ministry advocates for strict adherence to prescription-based medication; Aims to reduce rampant over-the-counter antibiotic use.
  - Prescription Documentation Advocacy; Ministry promotes obtaining antibiotics only through medical prescriptions; Requires proper documentation on a prescription.
  - Contrast with Over-the-Counter Practice; Ministry discourages self-diagnosis and verbal antibiotic requests at pharmacies; Promotes prescription-based medication
  - Ministerial Initiative on Rational Prescription; Minister of Health advocates for rational antibiotic prescribing; Focus on ensuring qualified personnel issue prescriptions."
  - Government Initiatives on Rational Prescription; Ministry advocates for strict adherence to prescription-based medication; Aims to reduce rampant over-the-counter antibiotic use.
- Interview 11
  - "Lagos State Government’s Efforts in Healthcare Improvement; Focus on strengthening primary healthcare institutions; Commissioner for

Health in Lagos State works on AMR-related policies."

- Interview 12
  - "Government Intervention in Healthcare; Strengthening Primary Healthcare; Urban-Rural Healthcare Disparities; Doctor Shortage in Rural Areas."
  - "Healthcare System Restructuring; Dialogue with Minister of Health; Importance of Primary Healthcare; Healthcare Reform Efforts."
  - Federal and State Healthcare Responsibilities; Strengthening Primary Healthcare as a Priority.
  - Government Oversight in Rural Healthcare; Strengthening Primary Healthcare; Challenges in Regulating Village Clinics; Inaccessible and Remote Healthcare Settings
- Interview 14
  - Government-Private Sector Collaboration During COVID-19; Successful Model for Wide-Scale Testing.
  - Government-Private Sector Collaboration in Health; Expanding Reach Through Private Facilities and Laboratories; Ongoing Discussions on Engaging Private Sector in AMR Policy

- Current AMR Surveillance Systems
  - Data Collection & Surveillance enhances tracking of antibiotic use, resistance trends, and stewardship impact to inform better AMR policies.
    - Interview 1
      - "Nigeria has conducted AMR surveillance since 2017; not yet optimal or fully representative but progress is underway."
      - Nigeria submitted AMR data from 2017–2022; issues with central site and reference lab prevented 2023 GLASS submission; working to improve data collection
    - Interview 2
      - "Agreed to integrate data from different disease entities to track resistant pathogen trends; includes bacterial, fungal, and viral resistance (HIV, TB);

        - AMR Net is a WhatsApp platform for facility networking; facilitates data and resource sharing; provides updates on national and global training and funding opportu.
        - Five facilities receiving funding; monitor progress; support action plan development and implementation; facilities conduct point prevalence surveys to track antibio
    - Interview 5
        - WHO recently partnered with Peishan to support primary healthcare, focusing on capacity building, standard data reporting, and recording; initiative started about a year ago
        - Some tertiary and secondary private hospitals conduct testing, but many do not fully engage in antimicrobial stewardship
        - "Partners like Commonwealth Partnership, Medical M-Taps USA, and CFID support AMR programs in hospitals, schools, and communities, but private sector involvement remains low."
        - "Held initial meetings to involve private sector in the National Action Plan, especially stewardship; church-affiliated hospitals follow stewardship practices and conduct testing."
        - AMR surveillance system includes diagnostic testing for febrile and suspected infection cases; private hospitals conduct bacterial identification and filtering
        - Some private hospitals have diagnostic facilities and conduct testing, but they are not included in the national AMR surveillance network; testing is done mainly for internal hospital use.
        - WHO actively supports stewardship and surveillance; some hospitals have AST (Antimicrobial Susceptibility Testing) facilities and conduct testing regularly.
        - Plan to enroll hospitals with AST facilities into the AMR surveillance system; goal is to expand data collection and generate evidence, as current surveillance sites are limited
    - Interview 6

        - "Antibiotics are often prescribed at the primary healthcare level; regulation is improving as data from the public sector is being analyzed to inform practices
        - Strengthening the AMR surveillance system to include private practitioners, alongside primary healthcare, government-owned facilities, and tertiary centers; ensuring full data integration
    - Interview 8
        - Antimicrobial Susceptibility Testing (AST) Practices; Both private and public hospitals conduct some level of AST; Surveys provide insights into current AST implementation
    - Interview 12
        - Antibiotic Stewardship Book; Ministry of Health Guidelines; Monitoring Antibiotic Use in Clinics.
    - Interview 14
        - "AMR Studies and Baseline Assessments; Antimicrobial Stewardship in Tertiary Hospitals; Capacity Evaluation of Stewardship Programs in Nigeria."
        - "Private Sector AMR Stewardship Programs; Second Phase of Fleming Fund Country Grant; Support for Establishing Stewardship in Private Healthcare."
        - "Engagement of Private Sector in AMR Stewardship; Recognition of Private Sector’s Role in Healthcare Access; Ongoing Efforts to Involve Private Sector in AMR Programs."
        - "Implementation of Point Prevalence Surveys in Hospitals; Understanding Antibiotic Use Patterns; Ongoing Efforts to Improve Monitoring of Antibiotic Usage."
        - "Point Prevalence Surveys in Tertiary Hospitals; Reflecting Highest Level of Care in the Healthcare System; Lower Levels of Care in Other Healthcare Facilities."
        - "Common AMR Stewardship Strategies; Prospective Audits and Point Prevalence Surveys; Estimating Patterns of Antimicrobial Use; Consistency in Conducting Point Prevalence Surveys."

                - "Lack of Approval Requirements for Senior Clinicians Prescribing Restricted Antibiotics; Increased Frequency of Point Prevalence Audits; Development of Treatment Guidelines in Hospitals."
                - "Increased Frequency of Point Prevalence Audits; Development of Treatment Guidelines in Hospitals; Creation of Facility-Specific Antibiograms."
- National and Global AMR Initiatives
    - Nigeria is a strong target for intervention due to ongoing national action plans, stewardship programs, and global partnerships combating antimicrobial resistance.
        - Interview 1
            - "National Action Plan outlines planned activities; Nigeria's healthcare delivery is categorized into primary, secondary, and tertiary levels;
            - AMR Network "Only two private hospitals in the AMR network, Babcock University Teaching Hospital and Limi Children's Hospital; Babcock excels in stewardship
            - "Implemented an integrated stewardship program based on literature recommendations; adopted as a national approach."
            - AMR Community of Practice; Virtual community of practice platform for facilities; shares ideas, events, and updates on AMR trends locally and globally.
        - Interview 2
            - "National action plan includes engagement of all healthcare levels, both private and public."
            - AMR Net is a WhatsApp platform for facility networking; facilitates data and resource sharing; provides updates on national and global training and funding opportu.
            - Five facilities receiving funding; monitor progress; support action plan development and implementation; facilities conduct point prevalence surveys to track antibio
            - Nigeria participates in the Prescribing Companion (CPA); app provides prescription

guidelines, national action plans, and stewardship resources; regularly updated.
    - Trained healthcare workers to implement antimicrobial stewardship in 52 hospitals; coverage remains limited given Nigeria’s size and needs
- Interview 4
    - Each institution has an Infection Prevention and Control Committee that develops prescription guidelines; stepwise approach prioritizes simpler antibiotics before stronger ones
    - Nigeria established the National Casualty Health Institution Standard Committee to monitor healthcare institutions and address key challenges
- Interview 5
    - "National Action Plan 2.0 (2023) developed using multi-level data; AMR remains a global top priority."
    - "Nigeria integrated WHO classification into its National Essential Medicines List; currently in finalization and will be published soon."
    - "National Action Plan developed using WHO's 6-step handbook; WHO is a member of Nigeria's National Pharmacy, Ethical, and Antibiotics Committee."
    - "WHO supports procurement and supply chain of antibiotics, vaccines, and diagnostics; committee member assisting in guideline implementation and development."
    - "Nigeria adopted WHO AWARE categorization; supporting expansion and scale-up of antimicrobial stewardship in hospitals."
    - WHO is a key technical agency in Nigeria's AMR response; provided technical and financial support for developing the National Action Plan over the last two years
    - Stewardship expansion started with tertiary hospitals, now moving to secondary and primary levels; 35 hospitals receiving technical support for guideline development

- WHO recently partnered with Peishan to support primary healthcare, focusing on capacity building, standard data reporting, and recording; initiative started about a year ago
- National Action Plan is finalized and awaiting launch; plan to revamp national technical working groups to improve functionality and include the private sector.
- National Action Plan 2.0 includes plans to revamp technical working groups and address private sector gaps
- Private initiatives like Peishan are emerging to revitalize primary healthcare, despite the primary focus on the public sector.
- Nigeria is in the foundational stage of its AMR program, developing stewardship systems, guidelines, and structures before moving into full implementation
- AMR guidelines and training modules have been developed but not yet published or launched; final stages of completion to support stewardship implementation
- Once enrolled, private sector facilities will follow national AMR guidelines; capacity building, mentorship programs, and data collection will be extended to the private sector.
- "WHO AWARE classification integrated into Nigeria's national standard treatment guidelines."
- "WHO supports the Ministry in developing stewardship guidelines and toolkits; recently developed a mentorship guide with partners; stewardship program needs expansion."
- Current AMR program is progressing well; Honorable Minister launched a sector-wide approach under health security to scale up access to diagnostics and medicines
- Honorable Minister's sector-wide approach presents a new opportunity for AMR integration, but not yet finalized

- WHO is taking initial steps to involve military and other hospitals in AMR stewardship, assessing data and capacity before expanding their role; collaborating with CHAN and other organizations to increase private sector involvement

- Interview 6
  - Private sector will be engaged as part of opportunities to include them in antimicrobial stewardship efforts through collaborative methodologies and ongoing initiatives
  - Policies for transparency and reporting lines are being enforced, including auditing HMO providers for antimicrobial stewardship as part of ongoing efforts to improve accountability
- Interview 7
  - Guidelines for AMR available at hospital level and from the Ministry of Health. Use of Essential drug list provided by the Ministry.
  - Prescription policies; Focus on generic drugs, pharmacy provides lists of available and out-of-stock drugs
  -
- Interview 8
  - UK Government Grant Flemming Fund, human health lead; address challenges of AMR
  - Willingness of Private Sector to Adapt; Private healthcare providers are open to new information; Receptive to initiatives that improve healthcare provision
  - Importance of Stewardship Committees; Establishing AMR stewardship committees in secondary and private facilities; Supports responsible antimicrobial use and monitoring
- Interview 9
  - Ministry of Health as a Communication Channel; Ministry can communicate directly with all doctors in Nigeria through hospital heads; Ensures nationwide awareness of AMR policies and best practices
- Interview 10

        - Ensuring Compliance with Antibiotic Guidelines; Members are educated on correct antibiotic use; The association enforces adherence to proper guidelines
        - "Educational Initiatives on Antimicrobial Stewardship; Committee engages with members through hospital visits, seminars, and awareness programs."
        - Ensuring Compliance with Antibiotic Guidelines; Members are educated on correct antibiotic use; The association enforces adherence to proper guidelines
    - Interview 11
        - "PNP's National-Level Collaboration on AMR; Engaged in joint efforts with the Nigerian Medical Association and other medical stakeholders."
        - "Past Partnerships on AMR Initiatives; Collaborated with the Lagos State government on antimicrobial resistance efforts."
        - "PNP's Collaborative Efforts on AMR Prevention; Partnered with the Nigerian Medical Association and Lagos State government; Engaged with other relevant organizations."
        - "Private Sector Partnerships for AMR Prevention; Collaborated with private organizations to advocate for antimicrobial resistance prevention."
        - "PNP's Partnership with Laboratories on AMR; Collaborated with Planar Lancet and other labs."
        - "PNP's Collaboration with Clinical Laboratories; Partnered with multiple clinical labs, including Planar Lancet."
    - Interview 12
        - Antibiotic Stewardship Book; Ministry of Health Guidelines; Monitoring Antibiotic Use in Clinics.
    - Interview 13
        - "Ministry of Health Handbook on Antimicrobial Resistance; Collaboration with the Center for

Disease Control in Nigeria; National AMR Policy."
        - "Opportunities for Antimicrobial Stewardship; Ministry of Health's Strategic Framework; Current Leadership's Focus on AMR Areas of Interest."
        - Policy Development on Drug Prescription; Chairman of Organization Leading Committee on Drug Prescription; Mandatory Doctor's Prescription for Dispensing Medications
    - Interview 14
        - AMR Research Projects in Northern Nigeria; Surveillance of Children with Fever; Microbiological Testing and Blood Cultures; Investigating Multi-Drug-Resistant Bacteria
        - "Focus on Antimicrobial Resistance (AMR); Research on Multi-Drug-Resistant Infections in Children; Personal and Organizational Commitment to AMR."
        - "AMR Research Projects in Northern Nigeria; Surveillance of Children with Fever; Microbiological Testing and Blood Cultures; Investigating Multi-Drug-Resistant Bacteria."
        - "Application of AwaRe Classification in Nigeria; Review of Antibiotics in the Essential Medicine List; Involvement in Adaptation of AwaRe for National Use."
        - "Capacity Assessment for AMR Stewardship Programs; Evaluation of Hospitals Without Stewardship Programs; National AMR Program Supported by Fleming Fund; Establishment of AMR Stewardship Programs in Pediatric Hospitals."
        - "Collaboration with Pediatricians in Nigeria; Member of Nigerian Society for Pediatric Infectious Disease; Setting Up AMR Stewardship Programs in Pediatric Units."
        - "Early Stages of Implementation; Growing Adoption of Stewardship Programs Across Hospitals."
        - "Private Sector AMR Stewardship Programs; Second Phase of Fleming Fund Country Grant;

Support for Establishing Stewardship in Private Healthcare."

- "Engagement of Private Sector in AMR Stewardship; Recognition of Private Sector’s Role in Healthcare Access; Ongoing Efforts to Involve Private Sector in AMR Programs."
- "Essential Medicine List Committee in Nigeria; Review of Essential Medicines for the Handbook; Role of the Ministry of Health in Drug Selection."
- "Adapting WHO AwaRe Classification to Nigeria's Essential Medicine List; Review and Categorization of Antibiotics; Restricting Overuse of Third-Generation Cephalosporins."
- "Importance of WHO AwaRe Classification for Nigeria’s Essential Medicine List; Policy Development for Antibiotic Categorization; Implementing AMR Policies in Healthcare."
- "Summary of Baseline Assessments; Antimicrobial Stewardship Capacities in 9 Tertiary Hospitals; Regional Distribution of Hospitals Assessed."
- "Findings from Baseline Assessments; Active AMR Stewardship Programs in 2 of 9 Hospitals; Lack of Support for Stewardship Program Implementation in 7 Hospitals."
- "Partial Implementation of AMR Stewardship Programs; Insufficient Training for Stewardship Committee Members; Lack of Clear Terms of Reference for Stewardship Committees."
- "Improvement in AMR Stewardship Programs; Increased Training for Stewardship Committee Members; Progress in the 2 Centers with Initial Programs."
- "Grant from Commonwealth Pharmacists Association; UK Fleming Fund Support for AMR Stewardship; Capacity Building for Pharmacists in Low- and Middle-Income Countries."
- "Initiation of AMR Stewardship Programs in 2 More Hospitals; Success in Implementing Stewardship Programs; Fleming Fund Commonwealth Pharmacist Association Grant."

            - "Common AMR Stewardship Strategies; Prospective Audits and Point Prevalence Surveys; Estimating Patterns of Antimicrobial Use; Consistency in Conducting Point Prevalence Surveys."
            - "Lack of Approval Requirements for Senior Clinicians Prescribing Restricted Antibiotics; Increased Frequency of Point Prevalence Audits; Development of Treatment Guidelines in Hospitals."
            - "Increased Frequency of Point Prevalence Audits; Development of Treatment Guidelines in Hospitals; Creation of Facility-Specific Antibiograms."
  - Strategic actions
    - Implementing Incentives for Practitioners
      - Offering accreditation, recognition, and career benefits can motivate physicians and healthcare workers to engage in antimicrobial stewardship.
        - Interview 5
          - State-level efforts focus on gaining private sector buy-in by linking accreditation and license renewal to compliance with reporting and AMR regulations, ensuring full participation
          - Private sector buy-in can be achieved through policy linkage to accreditation and license renewal, but success depends on strong advocacy and consistent engagement
        - Interview 6
          - "A reward-and-sanction system tied to accountability will ensure private sector facilities understand the importance of antimicrobial stewardship, driving meaningful participation."
          - "A naming-and-shaming or recognition system within the reward-and-sanction framework can motivate facilities to comply with reporting standards by publicly recognizing or penalizing adherence to AMR policies."
        - Interview 9
          - Strategy to Limit Indiscriminate Prescription; Increase the number of qualified health workers

(doctors, pharmacists); Ensures better access to healthcare and reduces misuse.

        - Interview 10
            - Strengthening AMR Stewardship with Resources and Enforcement; Providing resources and encouragement can improve guideline adherence; Government and regulatory bodies must enforce standards
        - Interview 12
            - "Retention of Resident Doctors; Advocacy for Better Salaries and Welfare; Medical Brain Drain to Europe, U.S., and Canada; Potential Return Programs for Nigerian Doctors."
            - Healthcare System Stability; Bridging the Gap Between Third and First World Standards; Retaining Medical Professionals in Nigeria; Strengthening the Healthcare Workforce
            - Expanding Healthcare Workforce; Benefits for Public and Private Sectors; Strengthening Rural Healthcare; Increasing Health Awareness Opportunities
        - Interview 13
            - "Developing AMR Champions in Private Health Institutions; Offering Incentives and Recognition Awards for Antimicrobial Stewardship Leadership."
            - "Non-Monetary Incentives for AMR Leadership; Recognition as Motivation for Improved Practices in Private Health Institutions."
        - Interview 14
            - Incentives for Private Sector AMR Stewardship; Offering Endorsements and Certifications for Proper Antibiotic Use; Encouraging Proper Patient Evaluation in Private Facilities.
            - Mandatory Refresher Courses for Clinical Personnel; Regulatory Requirements for Annual Practice Licenses; Enhancing Private Facility Standards Through Continuous Training.
- Encouraging Government Leadership
    - Interview 3
        - Financial constraints lead patients to buy incomplete treatments; health insurance could ensure full-course

    - medication access; government has a major role in addressing affordability.
    - Health insurance expansion could help control medication use and ensure proper dosing; reducing out-of-pocket spending can curb antimicrobial resistance by making full-course treatments more accessible
- Interview 4
    - "Strengthening primary healthcare can solve 60% of Nigeria's health issues; capacity building, supervision, and regulation are key; current administration forming an implementation plan."
    - "Capacity building, law enforcement, and monitoring at the constituency level are crucial for strengthening healthcare regulation."
    - Effective coordination can help mitigate AMR; the National Council on Health is Nigeria's highest decision-making body; its decisions are binding nationwide
    - Provide laboratory chemicals and materials at a lower cost to improve accessibility
    - Strengthen private sector capacity through training, equipment, and affordable lab consumables to enhance Nigeria’s AMR response
    - Expand health insurance coverage to ensure affordable access to proper healthcare; reduces financial barriers and encourages patients to seek treatment from reputable sources
- Interview 6
    - Identifying key stakeholders, such as community pharmacists, who have worked closely with the government, is crucial for engaging the private sector in AMR efforts
    - High political will is crucial for passing laws, especially to help private facilities comply with antimicrobial resistance efforts
    - Political will is key to implementing systems like doctor and nurse registries, both for public and private sectors, to improve antimicrobial resistance efforts
- Interview 8
    - "Government Collaboration for Structured Prescribing; Work with the government to develop prescribing strategies."

  - "Community and Government Support for Disease Prevention; Organizations and government aid in preventing disease outbreaks."
  - "WHO Support for AMR Efforts; WHO is ready to support initiatives reducing indiscriminate prescribing; Promotes responsible antimicrobial use."
  - Regulating Over-the-Counter Antibiotic Sales; Implementing controls on what medications can be bought without prescriptions; Helps reduce indiscriminate antibiotic use
- Interview 9
  - "Pharmacy Staffing Standards; Government mandates that every hospital should have at least one trained pharmacist; Compliance with staffing standards remains a challenge."
- Interview 10
  - "Government intervention and legislation needed; Proper regulation can improve the system."
  - "Regulations exist but are not enforced effectively."
  - "Federal Ministry of Health must enforce regulations; Street vendors openly sell antibiotics without prescriptions."
  - "Authorities must take action against illegal drug sales; Doctors lack prosecuting power and serve only as guides."
  - "Engage stakeholders in education and regulation; Emphasize long-term benefits of rational antibiotic use; Prosecute those violating regulations."
  - "Advocates for prosecution as a deterrent; Belief that strict measures are necessary for compliance in Nigeria."
  - "Setting legal examples will encourage compliance; Expected to reduce irrational antibiotic use."
- Interview 11
  - Importance of involving the government in AMR initiatives; Advocate for laws to guide healthcare providers, especially private practitioners; Government support is crucial for AMR regulation and implementation.
- Interview 12
  - "Regulation of Drug Production; Penalties for Importing Substandard Medications; Life Sentences for

Counterfeit Drug Manufacturers; Reducing Drug Abuse and Antibiotic Resistance."
        - "Strict Consequences for Fake Drug Importation; Imprisonment for Counterfeit Drug Traffickers; No Option for Fines."
        - Opportunities for Antimicrobial Stewardship; Improving Prescriber Knowledge; Government Enforcement of Medical Ethics; Increased Oversight by the Ministry of Health
        - "Accountability in Healthcare; Consequences for Medical and Pharmaceutical Actions; Importance of Funding Allocation."
        - "Increased Healthcare Budget Allocation; Federal and State Government Implementation; Retention of Medical Professionals; Reducing Dependence on Untrained Practitioners."
        - Low-Cost Healthcare Policies from Developed Nations; Potential Impact of Policy Enforcement on Antimicrobial Resistance; Changing the Pathway of AMR in Nigeria
    - Interview 14
        - Certifications and Endorsements for Private Healthcare Facilities; Improving Facility Ratings Through AMR Stewardship; Enhancing Patient Trust and Competitiveness
- Enhancing AMR Monitoring Systems
    - Strengthening surveillance and data collection is essential for tracking AMR trends, identifying effective stewardship strategies, and ensuring evidence-based interventions through reliable monitoring and reporting techniques.
        - Interview 1
            - "Stewardship requires evidence, support, and guidelines; locally generated data is essential for informed prescribing and decision-making in hospitals
        - Interview 2
            - "Private sector AMR data should be integrated; stewardship and AMR surveillance data need triangulation."
            - Best strategy for improving antimicrobial stewardship is close monitoring in hospitals.
        - Interview 3

        - "Prescription Documentation and Traceability; Ensure prescription sheets include medical practitioner’s name and registration number; Enables proper documentation and accountability."
        - "Proper Prescription Documentation; All prescribed medications should be documented on a prescription sheet; Prevent informal practices like using random pieces of paper."
        - "Verbal Prescription Issue; Some doctors give verbal prescriptions instead of written ones; Practices like verbal prescriptions contribute to antimicrobial resistance."
    - Interview 4
        - Enforce electronic medical records (EMR) across federal tertiary institutions to improve data management and accelerate AMR mitigation nationwide
        - "Plan to simplify antimicrobial stewardship; supervise healthcare providers and transition to electronic medical records; utilize two data venues."
        - State-of-the-art facilities improve diagnostics compared to outdated analog systems
        - Strengthen quality control in medical laboratory science through evolving regulatory bodies to improve diagnostics.
    - Interview 5
        - Assessment needed to understand Nigeria's healthcare structure; government focus is mainly on the public sector, which faces challenges in the six building blocks of healthcare
    - Interview 6
        - "Strengthening the AMR surveillance system to include private practitioners, alongside primary healthcare, government-owned facilities, and tertiary centers; ensuring full data integration."
        - "Overcoming resistance to data reporting by educating private practitioners on the importance of AMR surveillance; leveraging and improving existing policies is essential."

- Addressing concerns about data reporting through awareness and advocacy; leveraging key stakeholders and gatekeepers in the private sector to ensure successful implementation
- "Accurate data on the number of private sector facilities is crucial for effective AMR management; it is important to assess registered vs. unregistered facilities and their level of engagement."
- "Creating a directory of private practices is essential to understand what each facility is doing in terms of AMR management and to identify unregistered facilities, ensuring better regulation."
- "Conducting a survey to create a directory of private facilities is essential for understanding the number and status of private practices, similar to the Primary Healthcare Assessment."
- "Duplication of the Primary Healthcare Assessment for the private sector is necessary to gather data on private facilities, their level of AMR implementation, and to regulate antimicrobial stewardship."
- "Mandating antimicrobial stewardship in all private sector facilities is crucial, including establishing a reporting line and ensuring that contact details are collected to track compliance."
- "Instituting monthly reporting for private facilities on antibiotics usage, procurement sources, and quality is essential. It should involve pharmacovigilance teams to ensure compliance."
- "Continuous engagement with private sector facilities through regular reporting and annual or biannual review meetings is essential to ensure ongoing collaboration."
- "Implementing a performance framework with accountability through regular reviews will ensure private sector facilities report their activities, allowing for tracking of compliance."

            - IPC and antimicrobial resistance are closely linked; as part of surveillance and supportive supervision, monitoring these aspects is now a key responsibility
        - Interview 8
            - Antimicrobial Stewardship and Monitoring; Responsible prescribing is part of antimicrobial stewardship; Monitoring usage ensures proper implementation
            - Pilot Study for AMR Intervention; Conducting a small pilot can provide insights; Pilot projects can help scale interventions to wider community settings.
            - Complementary Strategies for AMR Mitigation; Pilot study complements practitioner training; Combining facility sampling and education strengthens AMR interventions
        - Interview 12
            - "Role of Guidelines in Proper Medication Use; Community Leadership in Curbing Drug Abuse."
        - Interview 13
            - "Strategy for Strengthening AMR Policies; Reviewing Outdated Policies; Involving Stakeholders in Policy Updates; Collaboration Between Regulators, Researchers, and Private Sector."
            - "Focus on Drug Prescription Policies; Preventing Antibiotic Abuse; Urgency in Addressing AMR Prescription Policies."
- Strengthening Regulatory Enforcement
    - trengthening enforcement of existing regulations ensures accountability, reduces antibiotic misuse, and promotes responsible prescribing practices to combat antimicrobial resistance.
        - Strong government regulation and enforcement are crucial to controlling low-quality drugs, holding providers accountable, and ensuring effective antimicrobial stewardship.
            - Interview 5
                - Regulations on antibiotic access exist but are not enforced; free access at the

community level, with vendors selling drugs on the street.
  - "Capacity building, law enforcement, and monitoring at the constituency level are crucial for strengthening healthcare regulation."
  - Improving regulation and legislation requires assessments to identify gaps; guidelines exist but need stronger implementation
- Interview 6
  - Oversight and regulation will drive the private sector to comply, ensuring accountability and transparency in reporting their activities
  - Focusing on primary prevention is key. If we prevent infections early, we reduce the need for antibiotics, thus limiting resistance. Strengthening infection prevention efforts will improve long-term AMR outcomes
- Interview 8
  - Regulating Over-the-Counter Antibiotic Sales; Implementing controls on what medications can be bought without prescriptions; Helps reduce indiscriminate antibiotic use
- Interview 10
  - "Government intervention and legislation needed; Proper regulation can improve the system."
  - "Regulations exist but are not enforced effectively."
  - "Federal Ministry of Health must enforce regulations; Street vendors openly sell antibiotics without prescriptions."
  - "Authorities must take action against illegal drug sales; Doctors lack prosecuting power and serve only as guides."

        - "Engage stakeholders in education and regulation; Emphasize long-term benefits of rational antibiotic use; Prosecute those violating regulations."
        - "Advocates for prosecution as a deterrent; Belief that strict measures are necessary for compliance in Nigeria."
        - "Setting legal examples will encourage compliance; Expected to reduce irrational antibiotic use."
    - Interview 12
        - "Regulation of Drug Production; Penalties for Importing Substandard Medications; Life Sentences for Counterfeit Drug Manufacturers; Reducing Drug Abuse and Antibiotic Resistance."
        - "Strict Consequences for Fake Drug Importation; Imprisonment for Counterfeit Drug Traffickers; No Option for Fines."
        - "Medical License Revocation as a Deterrent; Fear-Driven Compliance with Regulations; Ethical Enforcement Among Healthcare Professionals."
        - Medical Council of Nigeria Oversight; Disciplinary Actions Against Healthcare Professionals; Fear-Driven Compliance with Medical Ethics
        - "Strengthening Medical Accountability; Consequences for Malpractice; Clinic Closures and Asset Confiscation; Severity-Based Fines."
        - "Legal Consequences for Quackery; Sentencing of Unlicensed Practitioners; Stronger Enforcement in Cities; Need for Regulation in Rural Areas."
        - "Consequences for Quack Practitioners; Enforcement of Professional Boundaries; License Revocation for Unqualified Procedures."

            - "Deterrence Through Consequences; Cultural Impact on Compliance; Reducing Malpractice in Antibiotic Usage."
        - Interview 13
            - "Ethical Guidance for Practitioners; Ensuring Adherence to Professional Ethics; Registration and Oversight of Practitioners; Data Availability for Registered Practitioners."
- Building Multi-Sectoral Partnerships
    - Coordinating collaborative efforts across regulatory bodies, healthcare sectors, and stakeholders strengthens antimicrobial stewardship and AMR control.
        - Interview 1
            - "Encourage facilities to integrate stewardship-related committees; maximize resources and personnel for optimal implementation.
        - Interview 2
            - Encourage integration of IPC, Drug and Therapeutic Committees, and Pharmacovigilance programs to maximize resources and staff efficiency
        - Interview 3
            - Organized training and engaging more microbiologists to spread education could help address antimicrobial resistance challenges.
        - Interview 4
            - "Planning to launch the national action plan; private sector prioritized for inclusion in the next implementation phase."
            - Primary care plays a crucial role in AMR; many referrals happen after trial-and-error treatments; proper antibiotic use at this level can reduce unnecessary referrals.
            - "Plan to simplify stewardship; unite primary healthcare providers under one umbrella."
            - "Plan to simplify antimicrobial stewardship; ensure collaboration across all levels of government to oversee implementation and monitor progress."

        - Leverage traditional, religious, and political leaders to drive AMR awareness and compliance
        - Global support is essential; infections are not localized, highlighting the need for international cooperation in AMR efforts
        -
    - Interview 5
        - Private sector includes many medical professionals; unclear if they are engaged in stewardship; national technical working groups need revamping to enhance private sector engagement
        - Need to involve private sector at tertiary and secondary levels, including hospitals, clinics, and dispensaries in antimicrobial stewardship; currently not happening
        - Nigeria's large population (230M) and federal system require coordinated AMR efforts; collaboration needed across local, state, and national levels
        - Private sector involvement should begin with inclusion in the national technical working group to address their concerns, challenges, and provide targeted support.
    - Interview 6
        - Community pharmacists and patent medicine vendors were involved in previous AMR efforts, with a focus on tracking antibiotic sales and treatments; solid advocacy networks are needed.
        - "Engaged private sector contributors can act as spokespersons, encouraging others to align with government policies and regulations to continue practicing in accordance with AMR stewardship guidelines."
        - One Health approach involves cross-sectoral collaboration, including health and agriculture, to tackle antimicrobial resistance comprehensively.
        - Antimicrobial resistance (AMR) and stewardship require a multifactorial, multi-departmental

approach, engaging all key stakeholders across different sectors
        - Collaboration across sectors is essential for tackling AMR
        - Bringing everyone together, retraining, and including antimicrobial resistance in the curriculum and policy is key. Political willingness and strong governance are essential for long-term success.
    - Interview 8
        - Strategy for Antimicrobial Resistance Mitigation; Engage the private sector, which serves 60% of the population
        - Role of Community-Based Organizations; Community organizations are effective in raising awareness; Similar approaches can be used to educate on antimicrobial resistance
        - Leadership Role in Promoting Solutions; Educated facility leaders can advocate for antimicrobial stewardship; Leadership engagement supports resistance mitigation efforts.
        - Overcoming Resistance to Change; Experienced prescribers may be reluctant to adopt new practices; Education and WHO guidelines can encourage acceptance.
        - Preventing the Next Pandemic; Antimicrobial resistance is considered the next pandemic; Community leaders, stakeholders, and faith-based organizations play a role
        - "Influence of Religion and Community Leaders; Religious beliefs and community leaders impact healthcare decisions; Traditional and social structures influence prescribing practices."
        - "Educated religious and community leaders can help disseminate antimicrobial resistance awareness; Their influence can drive responsible healthcare practices in communities."
        - Leadership Involvement in AMR Efforts; Engaging healthcare facility leaders is crucial for

        success; Leadership buy-in ensures effective implementation of AMR strategies
        - Collaboration with Pharmacies and Chemists; Engaging pharmacies and chemists to regulate antibiotic dispensing; Encouraging responsible medication distribution
        - Regulating Over-the-Counter Antibiotic Sales; Implementing controls on what medications can be bought without prescriptions; Helps reduce indiscriminate antibiotic use
        - Opportunity for AMR Intervention; Engaging the private sector can reach 60% of the population; Strengthening private sector involvement presents a major opportunity.
    - Interview 9
        - Addressing AMR requires strategies at multiple levels; Solutions include employing more personnel, training, and enforcing legal actions for non-compliance
        - "Strengthening Stakeholder Collaboration for AMR; Involvement of Ministries of Agriculture, Health, Education, Environment, and Veterinary Doctors; Unified Approach to AMR."
    - Interview 10
        - "Collaboration among stakeholders is essential; Develop formulary policies to promote rational antibiotic use; Raise awareness on the importance of proper antimicrobial stewardship."
    - Interview 11
        - "Importance of Collaborative Efforts for AMR; Public-private partnerships are essential; Involvement of relevant stakeholders within and outside the healthcare sector."
        - "Achieving Common Goals in AMR Prevention; Collaboration with stakeholders inside and outside the healthcare sector; Focus on raising awareness about the common goal of AMR prevention."
        - "Inclusive Approach to AMR Prevention; Emphasizes collaboration across sectors to

achieve greater impact; Advocates for working beyond the healthcare space to address AMR."

    - "Expanding Advocacy Beyond Healthcare; Advocate outside the healthcare space to involve more stakeholders; Communicate the message of AMR prevention to professionals in other sectors."
    - Private institutions can collaborate with pharmaceutical industries and laboratories; Pharmaceutical giants play a crucial role in addressing antimicrobial resistance
    - "Partnership Opportunities with Pharmaceutical Giants; Private institutions can collaborate with major pharmaceutical companies like Novartis and GSK; Pharmaceutical collaborations can help improve antimicrobial stewardship.
    - Broad Range of Partnerships for AMR; Private sector can collaborate with laboratories, major organizations, and institutions; Partnerships with larger organizations can support AMR efforts.
    - Opportunities for Global and Local AMR Partnerships; Private sector can collaborate with global organizations like WHO and antimicrobial associations; Easy access to knowledge-sharing and funding sources
    - Private institutions and practitioners can collaborate with regulatory bodies in the antimicrobial sector; Numerous opportunities for partnerships in the fight against AMR
    - Key Strategies for AMR Prevention; Advocate for effective SOPs in collaboration with the State government; Focus on securing funding through collaborative proposals
  - Interview 12
    - Community Health Workers Filling Gaps; Improving Rural Healthcare Attractiveness; Need for Social Amenities; Better Remuneration for Rural Healthcare Workers
    - Supporting Rural Healthcare Workers and Families; Government Implementation and

Enforcement of Policies; Ensuring Effective Healthcare in Rural Areas.

- Interview 13
  - Involvement of Private Sector in Antimicrobial Resistance (AMR); Deficiency in Monitoring and Regulation of Private Healthcare; Public Institutions Playing a Larger Role in Regulation.
- Interview 14
  - "Education for Policymakers on AMR; Raising Awareness Among Policymakers about the Importance of AMR, Surveillance, and Stewardship."

Theme #4: Role of Patients

- Patient-related barriers
  - Harmful Patient Practices
    - How patient behaviors, including self-medication and non-adherence, contribute to antimicrobial resistance.
      - Interview 1
        - "Before prescribing antibiotics for suspected infection, practice sending patients for culture. Patients are anxious when waiting, insisting they are prescribed something."
        - "Patient compliance is an issue; will not complete antibiotic instructions"
        - "Patient compliance is an issue; will give their antibiotics to others around them"
        - "Unavailability of prescribed antibiotic brands, must find alternatives; patients share leftover antibiotics, leading to abuse in the community"
      - Interview 2
        - "Patients prefer an antibiotic before the lab to reduce costs"
        - AMR is driven by human behavior; difficult to change prescriber habits; challenge in shifting to guideline-based, evidence-driven targeted treatment
        - "Anticipates some difficulty in changing long-standing habits of patients"
      - Interview 3

        - "Patients receive incomplete antibiotic courses; contributes to high AMR cases in healthcare facilities."
        - "Haphazard prescription patterns in Nigeria; patients obtain antibiotics from small shops and patent medicine stores without trained pharmacists."
        - "Patients self-medicate with partial doses; stopping early without completing treatment contributes to antimicrobial resistance."
    - Interview 4
        - Trial-and-error prescribing leads to antimicrobial resistance; a major issue in healthcare practice
    - Interview 5
        - Social and cultural factors contribute to AMR; lack of community awareness leads to rampant and irrational antibiotic use
    - Interview 6
        - Community healthcare workers often serve as facility heads and prescribe medications; high AMR rates in Nigeria linked to out-of-pocket healthcare spending, allowing self-medication.
        - Engagement is the first step in involving the private sector in AMR efforts, but the vastness of the sector and the number of people involved make it challenging to implement effective AMR programs.
    - Interview 7
        - Antibiotic prescribing decisions; Patient demand, alternative treatments (hydrogen peroxide), communication and decision-making with patients
        - Barriers to good prescribing practices; Religious beliefs affecting drug acceptance, faith-based healing
        - Barriers to good prescribing practices; Patient literacy level, cultural and religious factors affecting drug acceptance
        - Barriers to good prescribing practices; Religious beliefs affecting drug acceptance, faith-based healing

- Interview 8
  - Cultural factors influence prescribing practices; Growing technological access is increasing public awareness
  - Prescription Adherence and Duration; Prescriptions are given based on disease and treatment period; Patients are expected to follow prescribed duration for effectiveness
  - Follow-Up and Treatment Review; Patients should return to the prescriber if there is no improvement; Ensures appropriate adjustments to treatment
  - Need for Prescription Accountability; Monitoring prescriptions helps track medication use and prevent misuse; Addressing over-the-counter antibiotic sales without regulation
- Interview 9
  - "Factors Contributing to AMR; Indiscriminate and improper antibiotic use accelerates resistance; Bacteria adapt when prescriptions are not followed correctly."
  - "Impact of Low-Quality Antibiotics on AMR; Patients may develop resistance even when following prescriptions; Reduced potency of low-quality antibiotics contributes to treatment failure.
  - Federal Ministry of Health Intervention and Oversight; Ministry resolves issues in both public and private hospitals; Patients are expected to access antibiotics only through proper prescriptions
  - Self-Prescription Issue; Many individuals in Nigeria engage in self-prescription; Patients go directly to pharmacies without consulting healthcare workers, leading to improper antibiotic use.
  - "Self-Prescription and Improper Dispensing; Patients self-prescribe or rely on unqualified individuals for medication; Pharmaceutical stores may dispense drugs without proper prescriptions, contributing to antimicrobial resistance."

- Interview 10
    - "Some individuals refuse medication due to religious beliefs; Such cases are rare but still present."
    - "Lack of awareness, adherence to guidelines, and education; Guidelines exist but are not followed."
- Interview 12
    - Antimicrobial Resistance (AMR); Over-the-Counter Antibiotic Access; Role of Patent Medicine Stores and Pharmacies
    - Antimicrobial Resistance (AMR); Self-Medication and Community Antibiotic Sharing; Over-the-Counter Antibiotic Access; Patient Non-Adherence to Prescriptions
    - Antimicrobial Resistance (AMR); Patient Impatience with Treatment; Antibiotic Switching; Profit-Driven Sales in Pharmacies
    - "Antimicrobial Resistance (AMR); Incomplete Antibiotic Regimens; Unsupervised Antibiotic Switching; Accumulated Resistance Over Time; Patient Mismanagement."
    - "Religious Beliefs and Healthcare Decisions; Refusal to Seek Medical Help; Cultural Barriers to Healthcare Access."
    - "Differences Between Urban and Rural Healthcare; Challenges in Global Private Practice; Contrasts in Healthcare Access and Regulation."
- Interview 13
    - Self-Medication and Antibiotic Abuse; Over-the-Counter Antibiotics; Impact of Healthcare Resource Shortages; Developing Countries' Healthcare Habits.
    - Misuse of Antibiotics; Overuse and Incorrect Dosing Beliefs; Public Perceptions of Faster Recovery Through Higher Doses
    - Self-Diagnosis Through the Internet; Online Search for Antibiotics; Purchase of Medication Without Professional Guidance; Contribution to Antimicrobial Resistance

        - "Cultural Beliefs and Practices; Historical Influence on Antibiotic Use; Public Perceptions of Antimicrobial Medications."
    - Interview 14
        - "Pressure to Prescribe Antibiotics in Private Sector; Profit Motive Influencing Prescribing Practices; Patient Demands Impacting Clinician Decisions."
- Financial Constraints and Antibiotic Access
    - Financial constraints lead to self-medication, incomplete treatments, and cost-driven prescribing decisions.
        - Interview 1
            - "Patients prefer an antibiotic before the lab to reduce costs"
            - "Antibiotic choice guided by patient’s financial capability"
            - "Patient financial issues, avoid doctor consultation to directly buy the drug. Patients go to the doctor as a last resort."
        - Interview 2
            - "Financial barriers can be a challenge; important to adapt to a patient psyche for effective communication"
            - "Paucity of funds limits access to antibiotics; assist patients in need"
        - Interview 3
            - "Some private facilities are run by non-clinicians; profit often prioritized over antimicrobial stewardship; patients receive partial antibiotic courses based on what they can afford."
            - "Financial barriers are the primary challenge in the community sector; many patients cannot afford standard treatment and seek care at remote facilities staffed by less qualified personnel."
            - "Patients take any affordable drug due to financial constraints; may buy incomplete doses instead of full treatment."
            - Financial constraints lead patients to buy incomplete treatments; health insurance could ensure full-course medication access;

government has a major role in addressing affordability.

- Interview 4
  - Patients seek cheaper healthcare options, often compromising proper treatment; health insurance coverage is still developing; government working on solutions.
- Interview 6
  - Community healthcare workers often serve as facility heads and prescribe medications; high AMR rates in Nigeria linked to out-of-pocket healthcare spending, allowing self-medication.
  - Out-of-pocket payments create tracking barriers; lack of records for privately paid treatments and reliance on the National Insurance Scheme for some patients hinder data collection
- Interview 7
  - Barriers to good prescribing practices; Patient ability to pay, economic situation
- Interview 11
  - "Impact of Poverty and Ignorance on AMR; Economic downturns and poverty hinder proper healthcare access; Ignorance and poverty often go hand in hand, preventing access to quality treatment."
  - "Patient Challenges in AMR; Ignorance and poverty lead patients to seek improper care; Financial constraints cause reliance on chemists instead of proper healthcare services."
- Interview 12
  - Antimicrobial Resistance (AMR); Patient Impatience with Treatment; Antibiotic Switching; Profit-Driven Sales in Pharmacies
  - Private Healthcare Preference for Profitable Areas; Financial Challenges in Rural Healthcare; Irregular Payment for Services; Barriers to Healthcare Investment in Remote Regions
  - Medical Ethics in Diagnosis and Referral; Challenges in Proper Patient Referral; Financial Pressures in Healthcare Facilities; Overprescription Due to Operational Costs.

        - Barriers to Antimicrobial Stewardship in the Private Sector; Funding Challenges for Patients and Clinics; Financial Constraints in Healthcare Implementation.
        - Financial Barriers to Antimicrobial Treatment; Patient Inability to Afford Strong Antibiotics; Adjusting Treatment Based on Cost and Sensitivity Testing (MCS
        - Financial Motivations in Private Clinics; Overprescription for Profit; Extending Antibiotic Duration for Financial Gain
      - Interview 13
        - "Financial Barriers to Healthcare; Out-of-Pocket Expenses Leading to Self-Medication; Bypassing Trained Medical Practitioners for Unlicensed Practitioners or Pharmacists."
      - Interview 14
        - Challenges with Proper Investigations in Private Sector; Lack of Routine Cultures for Antibiotic Selection; Cost Considerations Impacting Clinical Decisions.
  - Low Public Awareness of AMR
    - Lack of awareness leads to antibiotic misuse, self-medication, and poor adherence to treatment guidelines.
      - Interview 1
        - "Awareness is an issue as patients must be convinced of importance of culturing before antibiotics"
      - Interview 2
        - "Patient abuse of antibiotics; use of erythromycin for malaria and salmonella resistance"
        - "Low awareness about antimicrobial usage in Nigeria"
        - "Importance of awareness that antibiotics are not always needed"
      - Interview 3
        - "Patients self-medicate with partial doses; stopping early without completing treatment contributes to antimicrobial resistance."
      - Interview 5

        - Social and cultural factors contribute to AMR; lack of community awareness leads to rampant and irrational antibiotic use
        - Community-level antibiotic overuse stems from lack of awareness and understanding of AMR and its consequence
        - Awareness of proper antibiotic use is low and needs improvement; private point prevalence surveys conducted in some hospitals, but scaling up in the private sector is needed
    - Interview 6
        - Limited awareness and training capacity are challenges, as only a few people per facility are trained, even within public sector facilities, hindering broader AMR education
    - Interview 7
        - Barriers to good prescribing practices; Patient literacy level, cultural and religious factors affecting drug acceptance
        -
    - Interview 8
        - Cultural factors influence prescribing practices; Growing technological access is increasing public awareness
    - Interview 10
        - "Lack of awareness, adherence to guidelines, and education; Guidelines exist but are not followed."
        - "Some individuals refuse medication due to religious beliefs; Such cases are rare but still present."
        -
    - Interview 11
        - Lack of Education on AMR; Many patients are unaware of antimicrobial resistance and its consequences; Some seek medication from chemists instead of healthcare facilities
        - "Patients purchase drugs from chemists due to financial constraints; Lack of access to proper healthcare, including diagnostic tests, contributes to improper use of antibiotics."
    - Interview 12

                ■ "Religious Beliefs and Healthcare Decisions; Refusal to Seek Medical Help; Cultural Barriers to Healthcare Access."
                ■ "Differences Between Urban and Rural Healthcare; Challenges in Global Private Practice; Contrasts in Healthcare Access and Regulation."
            ○ Interview 13
                ■ Poor Awareness and Implementation of AMR Policies; Weak Collaboration Between Ministry of Health and Ministry of Agriculture; Need for Improved AMR Collaboration.
            ○ Interview 14
                ■ Public Awareness on Antibiotic Use; Changing Mindsets on Antibiotics for Fever; Educating the General Public on Appropriate Antibiotic Treatment
                ■ "Cultural Beliefs and Practices; Historical Influence on Antibiotic Use; Public Perceptions of Antimicrobial Medications."
- ○ Strategies
    - ■ Awareness strategies are essential to reduce antibiotic misuse, improve AMR understanding, and strengthen antimicrobial stewardship efforts.
        - Interview 1
            - ○ "Government intervention needed for awareness; use social media, radio, TV, and religious houses to inform the population"
            - ○ "Counsel patients against antibiotic abuse; explain risk of tolerance; lack of awareness is a key issue"
            - ○ "Discourage unnecessary antibiotic use; emphasize need for proper investigation before starting activity"
            - ○ "Encourage testing before starting antibiotics; ensure patients complete the full prescribed course"
            - ○ "Building awareness; Information, Education, and Communication (IEC) strategies used"
            - ○ Private practitioners' role in explaining antibiotics, not forcing
        - Interview 2
            - ○ "Importance of explaining to patients the source of antimicrobial resistance; illness"
            - ○ "Importance of Encouraging patients to use antibiotics properly; avoid leftover antibiotics"

    - Encourage testing before starting antibiotics; ensure patients complete the full prescribed course
- Interview 3
    - Social media, radio, and electronic media are key for AMR awareness; jingles on radio stations can remind the public about antimicrobial resistance and stewardship
    - Develop educational materials like leaflets and posters for health facilities; strategically placed to reinforce training and promote guideline adherence
- Interview 4
    - Awareness is not low; increasing awareness in major institutions can further strengthen AMR prevention efforts
    - Leverage traditional, religious, and political leaders to drive AMR awareness and compliance
- Interview 5
    - Standard treatment guidelines need promotion at the subnational level to reduce antibiotic overuse and misuse; key priority is private sector involvement from planning stages
- Interview 6
    - Engagement is the first step in involving the private sector in AMR efforts, but the vastness of the sector and the number of people involved make it challenging to implement effective AMR programs.
    - Increased awareness of antimicrobial resistance is driving a focus on policy regulation, prompting active engagement and questions at meetings about the ongoing policy efforts
    - Enlightenment and awareness are key; integrating antimicrobial resistance into in-service training for all training institutes is important to raise awareness
- Interview 7
    - Preventing antibiotic abuse; Educating patients on correct usage, reviewing drug use during follow-up, challenges with patient literacy and follow-up
    - Preventing antibiotic abuse; Educating and improving communication with patients
    - Key strategies; Clinical experience, patient interaction
    - Awareness and knowledge in private sector; Monthly lectures from drug companies, reading materials

- Interview 8
    - Follow-Up and Treatment Review; Patients should return to the prescriber if there is no improvement; Ensures appropriate adjustments to treatment
    - Prescription Adherence and Duration; Prescriptions are given based on disease and treatment period; Patients are expected to follow prescribed duration for effectiveness
    - Spreading Awareness Through Healthcare Professionals; Educated healthcare professionals can cascade AMR awareness to patients and the community.
    - Private Sector's Role in AMR Mitigation; Educating private sector health practitioners increases awareness of AMR; Responsible prescribing in private healthcare settings.
    - Role of Community-Based Organizations; Community organizations are effective in raising awareness; Similar approaches can be used to educate on antimicrobial resistance
    - AMR in Agriculture and Community Awareness; Antimicrobial resistance affects both humans and animals; Farmers need education on the risks of indiscriminate antimicrobial use.
    - "Expanding Awareness Strategies; Awareness should extend beyond word of mouth; Utilize phones, TV, and radio to reach a wider audience in the community."
    - "Use radio, TV, and social media to disseminate AMR information; Expanding outreach through various media enhances public engagement."
    - "Targeted AMR Awareness Programs; Separate initiatives for practitioners and community members; Organize practitioner-focused training and community outreach."
    - "Traditional Awareness Methods; Pamphlets and leaflets have been used to convey health information; Printed materials can support AMR awareness efforts."
    - "Community Awareness Initiatives; Monthly jingles can reinforce AMR awareness; Workshops and public drives can enhance community engagement."
    - "Impact of Clear Public Health Messaging; Multi-platform communication improves adherence."

    - "Effective Media Campaigns in Outbreaks; Jingles and media advisories were effective during past outbreaks; Public health messaging influences behavior by emphasizing AMR risks."
    - Lessons from Ebola Outbreak; Extensive public awareness efforts were used during the Ebola outbreak
    - Rapid Dissemination of IPC Messages; Infection prevention and control (IPC) messages reached both healthcare professionals and the public
    - Encouraging Responsible Dispensing Practices; Pharmacies and chemists should inquire about patient history before dispensing antibiotics; Helps curb indiscriminate antibiotic use
- Interview 9
    - "AMR Awareness Strategy; Focus on sustained and widespread awareness of antibiotic abuse dangers; Aims to reduce misuse and improve responsible prescribing."
    - "AMR Awareness Strategy; Mass and sustained awareness through various media (electronic, print, social); Educate the public on the dangers of self-prescription and antibiotic misuse."
    - Addressing AMR requires strategies at multiple levels; Solutions include employing more personnel, training, and enforcing legal actions for non-compliance
- Interview 10
    - Training and Media Engagement for AMR Awareness; Organize regular antimicrobial stewardship training; Use mass media for public education beyond hospitals
- Interview 11
    - Focus on Advocacy for AMR Prevention; Belief in the power of advocacy to improve antimicrobial resistance prevention; Strong emphasis on public health initiatives.
    - Advocacy as a Key Strategy for AMR; Strong belief in using advocacy to improve public health and quality of life; Prevention emphasized as the most effective approach.
- Interview 12
    - "Strengthening Primary Healthcare; Community Engagement in Healthcare; Role of Media and Traditional Leaders in Health Education."

    - Role of Healthcare Workers in Public Education; Community Engagement to Reduce Antibiotic Resistance; Improving Quality of Life in Rural Areas
    - Community Leadership in Healthcare Advocacy; Engaging Religious, Village, and Political Leaders; Ongoing Efforts to Improve Healthcare; Hope for Policy Change
    - "Media Coverage for Drug Malpractice Awareness; Involving Religious, Traditional, and Political Leaders; Consequences for Malpractice."
    - Expanding Healthcare Workforce; Benefits for Public and Private Sectors; Strengthening Rural Healthcare; Increasing Health Awareness Opportunities
    - "Community Engagement for Antibiotic Stewardship; Role of Traditional and Religious Leaders; Addressing Widespread Drug Abuse in Nigeria."
    - "Role of Guidelines in Proper Medication Use; Community Leadership in Curbing Drug Abuse."
- Interview 14
    - "Education for Policymakers on AMR; Raising Awareness Among Policymakers about the Importance of AMR, Surveillance, and Stewardship."
