# Supplementary material for: Barriers and facilitators to antibiotic stewardship in Nigeria’s private healthcare sector: A qualitative interview study with national health and regulatory interest holders
Source: PLOS Glob Public Health. 2026 Jan 6;6(1):e0005731. doi: 10.1371/journal.pgph.0005731 (PMC12774337; doi:10.1371/journal.pgph.0005731)
Supplement: Attachment — Submitted filename: PGPH-D-25-02175_Response_to_Reviewers.docx [file pgph.0005731.s005.docx]

# PGPH-D-25-02175 - [EMID:c37a144149a7ec15]

Barriers and facilitators to antibiotic stewardship in Nigeria’s private healthcare sector: a qualitative interview study with key interest holders

## RESPONSE TO EDITOR’S AND REVIEWERS’ COMMENTS

| **Reviewer** | **Comment** | **Author’s Response** |
| --- | --- | --- |
| Reviewer #1 Response to Data Availability Question  Have the authors made all data underlying the findings in their manuscript fully available (please refer to the Data Availability Statement at the start of the manuscript PDF file)? | Response: No | We thank the reviewer for this comment. An initial Data Availability Statement was included in our original submission (see line 755). Following the reviewer’s feedback, we have added this existing statement to the start of the manuscript PDF file to ensure it is clearly visible and in line with PLOS Data Policy requirements. The Data Availability Statement now appears at line 54, directly after the Keywords section. |
| Reviewer #1  Title | Please specify which groups were interviewed and consistently refer to them as stakeholders to make the title clearer and more precise. | We thank the reviewer for this thoughtful suggestion. We acknowledge the importance of clarity and consistency in terminology. However, we intentionally used the term “interest holders” throughout the manuscript, including in the title, instead of “stakeholders”, to promote more inclusive language and avoid colonial connotations associated with the latter. The choice aligns with recent scholarship advocating for terminology that better reflects equitable engagement in global health research. To enhance clarity, we have revised the title to specify the types of groups interviewed while retaining the term “interest holders”. The new title reads:  “*Barriers and facilitators to antibiotic stewardship in Nigeria’s private healthcare sector: a qualitative interview study with national health and regulatory interest holders*.” |
| Reviewer #1  Abstract | Provide a rationale for why participants were purposively selected. | We thank the reviewer for this important comment. Participants were purposively selected to ensure representation from key national and regional institutions directly involved in medicine use, regulation, and stewardship in Nigeria. This allowed us to capture diverse perspectives from individuals with leadership or technical roles in professional associations, federal agencies, and technical bodies (as already noted in the original text). These individuals were best positioned to provide insights into systemic, organizational, and policy-level factors influencing stewardship feasibility in the private healthcare sector. We have revised the abstract to clarify this rationale and include additional detail, specifying that participants were selected *“for their expertise and organizational roles relevant to antibiotic use and regulation in Nigeria”* (lines 28–30). |
| Reviewer #1  Abstract | Improve how results are reported and explicitly link them to the SEM framework.  Clearly distinguish between barriers and facilitators, or present broader, overarching themes.  Refine the conclusion to avoid repetition and provide a concise, actionable call to action. | We thank the reviewer for this insightful and constructive feedback. We have carefully reviewed the abstract and believe it appropriately reflects the study’s focus, key findings, and structure within the journal’s word limits. The abstract presents the results in alignment with the Social-Ecological Model (SEM), as noted in the sentence: *“Codes were grouped into four thematic domains reflecting the roles of key actors (private sector, practitioners, government and health systems, and patients), and mapped to the Social-Ecological Model (SEM) to reflect individual, organizational, and policy-level influences.”* (lines 33-35). Barriers and facilitators are clearly differentiated in the abstract, with results presented in a structured manner, aligning with the study’s analytical framework. Similarly, the conclusion emphasizes actionable implications, stating that the study *“underscores the urgent need for targeted, context-sensitive stewardship strategies tailored to Nigeria’s private healthcare sector.”* (lines 45-46). |
| Reviewer #1  Introduction | Use one consistent term, either interest holders or stakeholders. | We thank the reviewer for identifying this point. As noted earlier, we intentionally used the term “interest holder**s”** throughout the manuscript, including in the title, abstract, and main text, to promote more inclusive language and avoid the colonial connotations associated with the term “stakeholders.” We have reviewed the manuscript to ensure that “interest holders” is used consistently and that “stakeholders” does not appear. |
| Reviewer #1  Introduction | Avoid repeatedly citing statistics (e.g., AMR burden globally and in LMICs, or private sector underrepresentation). Streamline to maintain focus and readability. | We thank the reviewer for this observation. While we agree that readability is essential, we believe the selected statistics provide important context regarding the scale and urgency of the AMR challenge in Nigeria and globally. We have reviewed the section to ensure that all figures mentioned directly support the study’s focus. |
| Reviewer #1  Introduction | Improve readability by using shorter paragraphs and reducing the number of references per paragraph. | We thank the reviewer for this helpful comment. We believe the current paragraph structure and referencing appropriately support the flow of the manuscript. The number of references per section was intentionally maintained to ensure comprehensive coverage of key foundational evidence for the study. |
| Reviewer #1  Methods | Explain why 15 participants were considered sufficient (e.g., data saturation, representation). | We thank the reviewer for this important comment. The sample size of 15 participants was determined based on the study’s aim to capture diverse perspectives from key national and regional institutions that play a role in antibiotic use and stewardship in Nigeria. This aligns with recommendations for qualitative interview studies focused on institutional perspectives, where 12-20 participants are typically sufficient when sampling is purposive. Thematic saturation was applied during data collection and analysis. The recurrence of key themes across interviews indicated that additional interviews were unlikely to yield substantially new insights. The research team remained on standby to conduct further interviews if saturation was not reached. |
| Reviewer #1  Methods | Table 1 is difficult to interpret; consider moving it to the appendix or summarizing participants by category and sample size. | We thank the reviewer for this comment. Table 1 was included in the main text to clearly present all organizations represented by interviewed participants. As the study explores perspectives from key national and regional institutions, listing these organizations in this table provides transparency about which organizations are represented. Keeping the table in the main text allows readers to easily reference the participating organizations while reading the Methods section. For this reason, we prefer to retain the current format unless the journal requires otherwise. |
| Reviewer #1  Methods | Clarify whether interviews were conducted in English only or if translation was required. | We thank the reviewer for this excellent point. In Nigeria, English is the official language and is commonly used in professional and institutional settings. Therefore, all interviews were conducted in English, and translation or interpretation was not required or offered. To clarify this in the manuscript, we have revised the sentence at line 128 to read:  *“Interviews were conducted remotely in English between 13 August 2024 and 13 December 2024, using Zoom (2025 Version 6.3.11) or Microsoft Teams (2025 Version 25031).”* |
| Reviewer #1  Methods | Describe any pilot testing or refinement of the interview guide during data collection. | We thank the reviewer for this helpful suggestion. The interview guide was developed collaboratively by the research team and reviewed to ensure relevance and clarity. While no formal pilot testing was conducted, the guide was refined iteratively during the initial phase of data collection based on early participant responses. Minor adjustments were made to improve question flow and clarity and ensure alignment with the study objectives. |
| Reviewer #1  Methods | Expand on researcher positionality and reflexivity: Were interviewers Nigerian? | We thank the reviewer for this important comment. The interviewers were not Nigerian but worked in close collaboration with our Nigerian research partners, who facilitated initial outreach and scheduling with participants. Importantly, the study’s co-PI and co-author is of Nigerian descent with over 15 years of experience in Nigeria's health sector, providing critical contextual expertise and cultural insight throughout the research process. All interviewers received training in global health research best practices, including culturally sensitive interviewing and reflexivity. Throughout data collection, the team engaged in regular reflexive discussions to contextualize findings and minimize interpretive bias. These practices were intended to ensure respectful engagement and accurate representation of participant perspectives. |
| Reviewer #1  Methods | Provide the dates and approval numbers for ethical review. | We thank the reviewer for this comment. The approval numbers for all ethics approvals are provided in the Ethical considerations section. Specifically, ethical approval was obtained from:   - The University of Ottawa Research Ethics Board (approval no. H-06-24-10154), - The Lagos State Health Research Ethics Committee (approval no. LSHREC/2024/00012), and - The Federal Capital Territory Abuja State Health Research Ethics Committee (approval no. FHREC/2024/01/138/13-06024).   These are highlighted in lines 160-168. Copies of ethics approval certificates from all these Research Ethics Boards indicating the date and duration of approval were also provided as part of our supplementary material, as required by the journal. |
| Reviewer #1  Methods | How might institutional affiliations (Ottawa vs. Nigeria) have influenced responses? | We thank the reviewer for raising this important point. While the interviewers were affiliated with Canadian institutions, the study was conducted in close collaboration with Nigerian research partners and co-authors who facilitated participant recruitment and engagement. This partnership helped establish trust and contextual relevance. We acknowledge that institutional affiliations may have influenced participant responses to some extent, and we have noted this as a limitation in the manuscript (lines 707-711):  “*Furthermore, the interviewers were affiliated with Canadian institutions and were not Nigerian, which may have influenced participant responses. To address this, interviews were organized in close collaboration with Nigerian research partners who facilitated recruitment and scheduling, and all interviewers received training in global health research best practices, including culturally sensitive interviewing and reflexivity.”* |
| Reviewer #1  Data Analysis | Clarify whether the coding-to-theme structure was developed inductively from the data or applied deductively. | We thank the reviewer for this question. As detailed in lines 155-156 of the Data analysis section, the coding process was primarily inductive, as descriptive codes were generated directly from participants’ responses and refined iteratively. These codes were then grouped into broader themes informed by the Social-Ecological Model, which provided a deductive framework for organizing the findings. This allowed us to remain grounded in the data while aligning the thematic structure with a well-established conceptual framework. We made some revisions to the section to clarify these aspects. |
| Reviewer #1  Data Analysis | The sequence from codes to subthemes to SEM is currently confusing; consider simplifying for clarity. | We thank the reviewer for this observation. The sequence from codes to subthemes to SEM reflects the structured progression of our analysis: codes were generated inductively from the data, grouped into subthemes representing key concepts, and then organized according to the Social-Ecological Model. We have revised the Data Analysis section to enhance clarity. |
| Reviewer #1  Data Analysis | Explain the combined use of NVivo and Excel, e.g., NVivo for coding and Excel for organizing/exporting results, to avoid perceived duplication. | We thank the reviewer for this comment. NVivo and Excel were used in complementary ways as part of a single analytic process, with no duplication of effort. NVivo supported the initial coding and thematic organization of interview transcripts, allowing us to systematically identify and refine descriptive codes. Microsoft Excel was then used to manage and structure the coded data across themes and subthemes, facilitating consistency in categorization and enabling clear visualization of patterns aligned with the SEM. |
| Reviewer #1  Results | Review the results for clarity, conciseness, and alignment with the study objectives. | We thank the reviewer for this thoughtful suggestion. We have carefully reviewed the Results section to ensure that the presentation of findings remains clear, concise, and directly aligned with the study objectives. The current structure reflects the key themes and insights identified through our analysis, organized by actor domain and mapped to the Social-Ecological Model. We believe this format effectively communicates the multi-level barriers and facilitators relevant to stewardship implementation in Nigeria’s private healthcare sector as emerged from the interviews. |
| Reviewer #1  Results | Ensure themes are clearly linked to the research questions and SEM framework | We thank the reviewer for this helpful comment. The Results section is organized according to the Social-Ecological Model (SEM), with themes reflecting the main actor groups and levels relevant to the study’s research questions. Specifically, each theme (private sector, practitioners, patients, and government and health systems) is mapped to one of the pertinent SEM levels (individual/interpersonal, organizational, or system/policy) and structured to highlight barriers, facilitators, and opportunities for antibiotic stewardship implementation in alignment with our research objectives. |
| Reviewer #1  Results | Revise subtheme associations to enhance comprehensibility. | We thank the reviewer for this suggestion. The current subtheme structure was intentionally designed to reflect the complexity of antibiotic stewardship in Nigeria’s private sector. Subthemes were organized to capture the interconnected dimensions (knowledge and perceptions, barriers, opportunities, and strategies) across each actor group (private sector, practitioners, patients, and government/health systems). This structure allows for a good comparison across levels of the Social-Ecological Model (SEM) and maintains clarity between participant perspectives. We are happy to consider further refinements and would welcome any specific suggestions the reviewer may have in this regard. |
| Reviewer #1  Results | Consider moving detailed tables to supporting materials and providing a summarized description of themes and - subthemes in the main text. | We thank the reviewer for this suggestion. Table 2 was included in the main text to provide readers with a clear overview of the themes and subthemes emerging from the interviews, organized by SEM level. Given the centrality of these findings to the study’s analytical framework, we believe retaining the table in the main text supports transparency and accessibility. However, we are happy to consider relocating it to the supplementary materials if the journal’s editorial team deems this preferable. |
| Reviewer #1  Discussion | The discussion is overly long and reference-heavy. Streamline by emphasizing what the study uniquely adds rather than summarizing multiple external studies. | We appreciate this thoughtful suggestion. We acknowledge the importance of maintaining focus on the study’s unique contributions. The current structure of the Discussion section was designed to situate our findings within the broader literature and highlight their relevance to ongoing antimicrobial stewardship efforts in LMICs. That said, we have reviewed the section to ensure that the emphasis remains on what this study uniquely adds - particularly the perspectives of national-level interest holders on stewardship feasibility in Nigeria’s private healthcare sector. |
| Reviewer #1  Discussion | Highlight new insights that were not previously documented and explicitly discuss how findings advance or challenge existing knowledge. | We thank the reviewer for this valuable recommendation. We have reviewed the Discussion to ensure that novel contributions of our study are clearly articulated. Specifically, our work is among the first to systematically explore national-level perspectives on antimicrobial stewardship in Nigeria’s private healthcare sector – a setting that remains underrepresented in AMR research and policy. The study highlights unique insights into institutional openness to stewardship, licensing-linked training mechanisms, and the role of regulatory partnerships, which have not been widely documented in previous literature. |
| Reviewer #1  Discussion | Strengthen policy and practice implications: specify what Nigerian policymakers and private providers should prioritize and what is feasible. | We thank the reviewer for this valuable feedback. Our discussion and conclusion sections emphasize several actionable priorities for both Nigerian policymakers and private healthcare providers. Participants underscored the need for *“improved training programs, broader and more effective use of guidelines and standard operating procedures, public-partner partnerships, and expanded awareness campaigns,”* (line 623-625) as well as *“political will, adequate funding, and stronger regulatory enforcement to address barriers such as limited antibiotic availability, poor laboratory infrastructure, and workforce shortages.”* (line 665-667). The paper also highlights the importance of *“more robust enforcement mechanisms and stricter penalties to deter non-compliance.”* (line 669-670). |
| Reviewer #1  Discussion | Acknowledge potential social desirability bias due to senior-level participants and clarify how the absence of frontline providers and patients limits interpretation of private-sector dynamics. | We thank the reviewer for this observation. We have added a statement to the limitation section acknowledging the potential for social desirability bias given that most participants held senior-level positions. Their perspectives may reflect institutional priorities rather than frontline realities. Additionally, we recognize that the absence of direct input from frontline providers and patients limits our ability to fully capture the day-to-day dynamics of antibiotic use in private healthcare settings, which was beyond the scope of our study. We have noted these limitations and emphasized the need for future research that includes these groups to complement our findings. |
| Reviewer #2  Discussion & Analysis | The paper reads well. However, the analysis is very surface level in the sense that the results read like a list. For example, the paper listed barriers but have not gone deeper into the analysis, looking at the differences between the narratives of the participants in terms of their work experience and responsibilities. For examples, the ones from the private sector, did they give different response in some areas compared to other participants? Also, the way barriers have been clustered together for providers and patients, it is not clear which are the most significant barriers, for example. The authors only present the barrier and the number of people reporting that. The discussion also does not talk about implications of the findings. The authors mostly compare their findings with other studies. | We thank the reviewer for this constructive feedback. As explained in the manuscript, the study was designed to capture perspectives from national- and institutional-level experts representing a range of professional roles and organizations. Our analysis was meant to emphasize patterns and intersections of these patterns across these different actor groups (private sector, practitioners, patients, and government/health systems). This approach aligns with the SEM, guiding both the coding and analysis by focusing on multi-level interactions that shape stewardship feasibility rather than the individual participant characteristics.  In the Results section, these differences are shown in how barriers and facilitators were organized thematically by actor level. For instance, we identified barriers such as “inconsistent prescribing practices, limited access to diagnostics, weak regulatory enforcement, and knowledge gaps among practitioners and patients,” and contrasted these with system-level constraints such as “workforce shortages and fragmented AMR surveillance infrastructure.” Likewise, facilitators and strategies were presented by level, showing differences and connections between actors.  Regarding implications, the Discussion and Conclusion sections explicitly outline practical and policy-relevant actions. These include “standardized training programs, public-private collaboration, stronger regulatory frameworks, performance-based incentives, and community-level awareness campaigns,” as well as the call for “targeted, context-sensitive stewardship strategies tailored to Nigeria’s private healthcare sector.” |
