# Supplementary material for: Barriers and facilitators to antibiotic stewardship in Nigeria’s private healthcare sector: A qualitative interview study with national health and regulatory interest holders
Source: PLOS Glob Public Health. 2026 Jan 6;6(1):e0005731. doi: 10.1371/journal.pgph.0005731 (PMC12774337; doi:10.1371/journal.pgph.0005731)
Supplement: Attachment — Submitted filename: PGPH-D-25-02175_R2_Response_to_Reviewers.docx [file pgph.0005731.s006.docx]

# PGPH-D-25-02175 - [EMID:c37a144149a7ec15]

Barriers and facilitators to antibiotic stewardship in Nigeria’s private healthcare sector: a qualitative interview study with key interest holders

## RESPONSE TO EDITOR’S AND REVIEWERS’ COMMENTS

| **Reviewer** | **Comment** | **Author’s Response** |
| --- | --- | --- |
| Journal requirements | If the reviewer comments include a recommendation to cite specific previously published works, please review and evaluate these publications to determine whether they are relevant and should be cited. There is no requirement to cite these works unless the editor has indicated otherwise.   Please review your reference list to ensure that it is complete and correct. If you have cited papers that have been retracted, please include the rationale for doing so in the manuscript text, or remove these references and replace them with relevant current references. Any changes to the reference list should be mentioned in the rebuttal letter that accompanies your revised manuscript. If you need to cite a retracted article, indicate the article’s retracted status in the References list and also include a citation and full reference for the retraction notice. | The reference list has been reviewed and small edits made to ensure accuracy. |
| Reviewer #2 | Remove the quotation in L706 from the discussion section. Not needed. We simply discuss in discussion section, not introduce any new result. It is okay to put quant data but we usually do not put qualitative data (quotes) in discussion. | Thank you very much for this helpful comment. We agree that qualitative quotations should not be introduced in the Discussion. The participant quote previously located in the Discussion section (line 682 in the revised manuscript) has now been removed, and the surrounding text has been revised to ensure that no new qualitative data are presented in this section. This change is visible in the tracked-changes version of the manuscript. |
